# Assisted evolution of corals and their symbionts enhances recruit heat tolerance but with complex outcomes

**DOI:** 10.1126/sciadv.aeb5575

**Published:** 2026-07-01

**Authors:** Annika M. Lamb, Guy A. McCutchan, Carys A. Morgans, Elizabeth A. Ivory, Murray Logan, Matthew R. Nitschke, Madeleine J. H. van Oppen

**Affiliations:** ^1^ Australian Institute of Marine Science - 1526 Cape Cleveland Road, Cape Cleveland, QLD 4810, Australia.; ^2^ Faculty of Science and Engineering, Southern Cross University, East Lismore, NSW 2480, Australia.; ^3^ School of Biological Sciences, Victoria University of Wellington, Wellington, New Zealand.; ^4^ School of Biosciences, University of Melbourne, The University of Melbourne, Parkville, VIC 3010, Australia.

## Abstract

Heat-tolerant coral stock could supplement populations with tolerance-conferring alleles to combat rising sea surface temperatures and marine heat waves. We tested coral selective breeding and Symbiodiniaceae experimental evolution independently and in tandem as interventions for generating heat-tolerant stock. Broodstock from two sites were ranked using Symbiodiniaceae photochemical efficiency under rapid heat stress and crossed to produce offspring from heat-tolerant colonies (top 25th percentile) and control offspring. Offspring were inoculated with heat-evolved or wild-type symbionts and exposed to 28°C (ambient) or 32°C (elevated) for 2 months. Selective breeding using rapid assays enhanced Davies but not Moore Reef recruit survival and growth at 32°C, suggesting that this method does not universally generate heat-tolerant coral due to genetics, maternal effects, and/or acclimation. Heat-evolved symbionts enhanced survival and bleaching resilience at 32°C but reduced growth at 28°C. Combining interventions yielded additive benefits, no enhancement, or resulted in one intervention diminishing the other’s impact. These results demonstrate assisted evolution’s potential while cautioning against generalizing its outcomes.

## Teaser

One size does not fit all: Assisted evolution enhances heat tolerance, but outcomes vary by trait, environment, and population.

## INTRODUCTION

Global biodiversity and the ecosystem services it provides are threatened by climate warming and the increased frequency, intensity, and duration of extreme weather events (*1*). This is because species face extinction when they cannot adapt, acclimate, and/or migrate at a rate that “keeps pace” with environmental change (*2*). The mass species extinction unraveling throughout the Anthropocene (*3*, *4*) could be partly mitigated through reductions in climate emissions that would slow and perhaps halt further climate change, buying time for species to adapt (*5*). While action is taken against climate change, management strategies that facilitate species adaptation will also contribute to the conservation of biodiversity (*6*–*8*).

Assisted evolution is a management approach that aims to assist the adaptation of species through increasing the frequency of alleles that confer resilience to climate change within populations (*9*–*11*). This might be achieved through selective breeding or laboratory selection applied to either the host organism or their microbial symbionts. These interventions aim to generate stock with enhanced climate resilience that might then be used to supplement populations with tolerance-conferring alleles (*11*–*13*).

Coral reef ecosystems harbor immense biodiversity and are particularly threatened by climate change induced heat waves that cause mass mortalities of reef-building corals (*1*, *14*). High temperatures drive coral bleaching, which is a breakdown in the symbiosis between the coral host and their Symbiodiniaceae and often results in mortality (*15*). Bleaching events in 2016, 2017, and 2020 resulted in estimated declines of 9.8, 5.5, and 11.8% in coral cover (*16*) and 26, 50, and 71% in larval supply (*17*), respectively, across the world’s largest reef: the Great Barrier Reef (GBR). Furthermore, the most recent 2024 mass bleaching event—compounded by additional stressors including cyclones, freshwater inundation, and crown-of-thorns starfish—resulted in coral cover declines of 14 to 30% among the GBR regions, with individual reefs experiencing losses of up to 70.8% (*18*). Globally, climate change is expected to overwhelm coral reef ecosystems such that reef refugia—areas unaffected by climate stress—are estimated to become extremely rare under a 1.5°C scenario and nonexistent under a 2.0°C scenario (*19*). In the face of this threat, there is a dire need for research and development of effective reef management strategies (*20*, *21*).

Reef-building corals and their symbiotic partners (collectively the coral holobiont) are the ecosystem engineers of corals reefs. The assisted evolution of the coral host and/or its microbial symbionts will therefore have amplified benefits to the conservation of coral reef biodiversity (*11*, *13*, *22*). Given the complexity of coral reef ecosystems and the projected increase in the intensity of summer heat waves (*1*), it is likely that multiple interventions will be required to achieve thermal enhancements that allow corals to survive these extreme events. It is theoretically possible for multiple interventions that target the same or different component/s of the coral holobiont to have additive and/or antagonistic effects and that the independent and combined effects of interventions might be context dependent. It is therefore important to test interventions independently and in tandem across environments and target species.

Selective breeding has been proposed as an intervention to generate coral stock with enhanced heat tolerance (*23*). Selective breeding involves conducting controlled crosses based on broodstock phenotypes and/or genotypes to produce individuals that are enhanced with respect to a desired trait. This approach has improved the yield and resilience of agricultural stocks (*24*–*26*), and conservationists have more recently considered selective breeding as a management tool (*27*, *28*). The polygenic nature of coral heat tolerance impedes the development of a genetic assay to identify heat-tolerant broodstock that could be used for selective breeding of this trait (*29*, *30*). However, phenotype-informed selective breeding has effectively generated corals with enhanced heat tolerance, albeit with mixed results that indicate the phenotype and heat stress conditions used to assay coral broodstock affect the success of this strategy (*23*, *31*). Rapid heat stress assays have been widely used to phenotype coral heat tolerance (*32*–*35*) and are an attractive tool for selective breeding for scalable restoration since they are high throughput. However, trait values typically measured using rapid heat stress assays (i.e., photophysiology) may be particularly influenced by nongenetic factors such as thermal history (*33*) and may primarily reflect symbiont rather than host genetics. It is therefore critical to assess the utility of rapid heat stress assays in selective breeding programs aimed at enhancing coral resilience under climate change.

Symbiodiniaceae-cultured ex hospite can be exposed to thermal selection (i.e., experimental evolution) to generate strains that are adapted to higher temperatures (*36*–*40*). The heat tolerance of the coral holobiont is affected by the Symbiodiniaceae community (*41*), and experimentally evolved strains have been used to inoculate larval, juvenile, and adult corals to improve their heat tolerance (*42*–*44*). While the deployment of heat-evolved Symbiodiniaceae (hereafter referred to as HE symbionts) constitutes a promising intervention, questions remain regarding how generalizable enhancements of heat tolerance in corals inoculated with HE symbionts are across coral species, genotypes, and life stages. Furthermore, trade-offs in corals inoculated with HE symbionts across traits and between environments must be explored to develop a wholistic understanding of the consequences of this intervention (*45*).

Here, we tested the efficacy of two assisted evolution interventions on coral recruits maintained in the laboratory in isolation and in tandem: (i) selective breeding of the coral host using phenotypes obtained via rapid heat stress assays and (ii) inoculation of recruits with HE symbionts. This research makes substantial contributions to the development of assisted evolution interventions that might be used in the conservation of biodiversity.

## RESULTS

### Broodstock heat tolerance

**Fig. 1.**
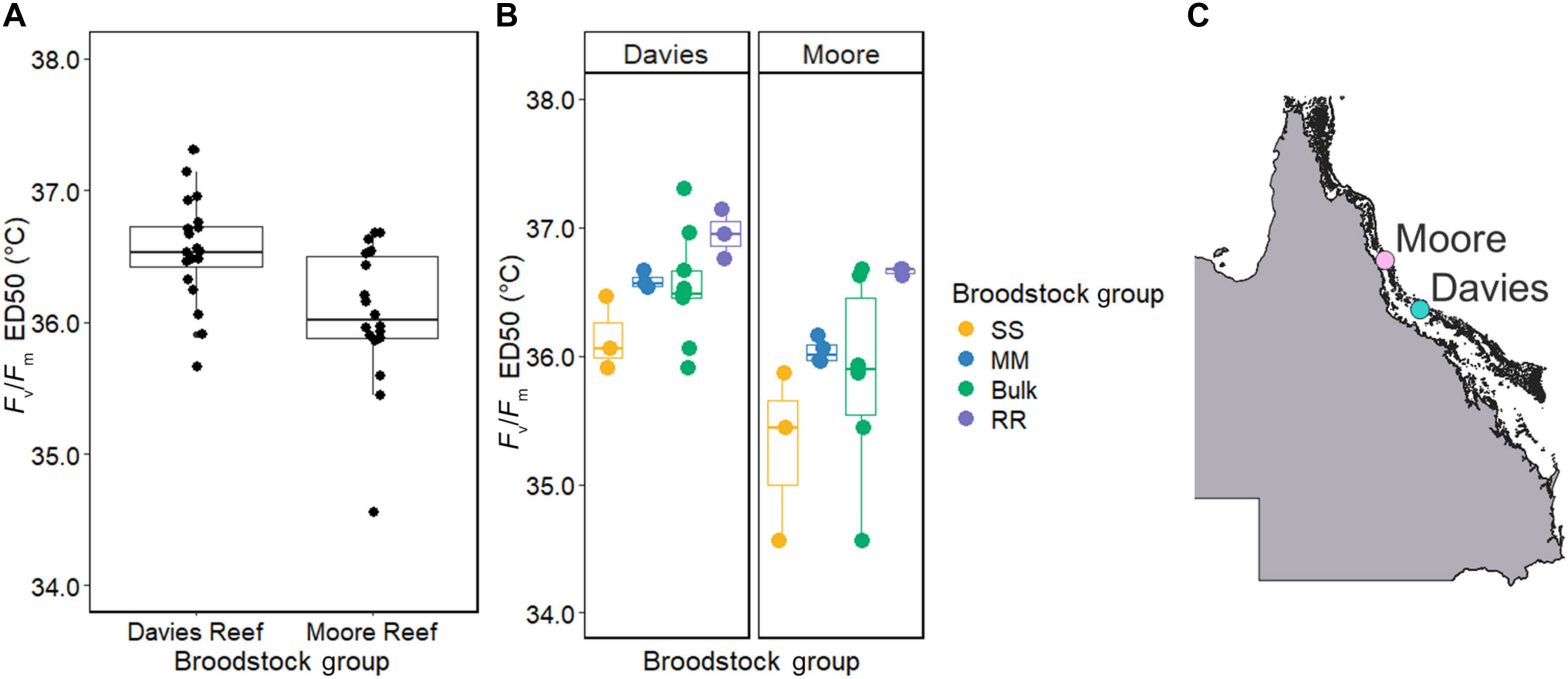
Performance and collection locations of coral broodstock. Panel (**A**) depicts the total distributions of *F*_v_/*F*_m_ ED_50_ of the tested populations, and panel (**B**) depicts the distributions of the parental colonies of each Moore Reef and Davies Reef offspring group. Panel (**C**) depicts the locations of Moore Reef and Davies Reef on the Great Barrier Reef. Offspring groups were produced from crosses among the most sensitive (SS), moderately resilient (MM), and most resilient (RR) broodstock colonies, and bulk crosses (Bulk) produced from all broodstock gametes spawned in synchrony. The horizontal lines of the boxes represent the lower quartile, median, and upper quartile values; the “whiskers” represent the extreme values; and dots represent the *F*_v_/*F*_m_ ED_50_ values of colonies. Larger ED_50_ values are interpreted as indicative of greater heat tolerance.

Rapid heat stress assays were conducted to test the heat tolerance of *Acropora spathulata* broodstock colonies from Davies and Moore Reefs on the GBR and inform selective breeding. Maximum quantum yield (*F*_v_/*F*_m_) ED_50_ (half-maximal effective dose) values were obtained using rapid heat stress assays and applied to selective breeding because *F*_v_*/F*_m_ has correlated with expected patterns of heat tolerance across locations in multiple species, including *A. spathulata* (*32*, *34*, *35*, *46*). An ED_50_ value constitutes the temperature that induces a 50% decline in *F*_v_/*F*_m_ in a colony compared to its *F*_v_/*F*_m_ at the maximum monthly mean (MMM) of the source reef, with higher values indicating greater heat tolerance (*35*). The variance of *F*_v_/*F*_m_ ED_50_ of the Moore Reef and Davies Reef broodstock was equal (*F* = 1.35, *P* = 0.252; Fig. 1A). Moore Reef broodstock had lower *F*_v_/*F*_m_ ED_50_ thresholds than the Davies Reef broodstock (difference in means = −0.49, 95% confidence interval = −0.19 to −0.80, *P* = 0.002; Fig. 1A), and the former was thus the more thermo-sensitive population.

The egg-sperm bundles of the broodstock were combined in bulk crosses according to broodstock heat tolerance (*F*_v_/*F*_m_ ED_50_ values) to produce four offspring groups for each reef: (i) resilient × resilient (RR) from the most heat-tolerant broodstock, (ii) moderate × moderate (MM) from moderately heat-tolerant broodstock, (iii) sensitive × sensitive (SS) from the most thermally sensitive broodstock, and (iv) bulk from all colonies that spawned on the night when the most colonies spawned in synchrony. Enough colonies released sufficient gametes in synchrony to generate the eight offspring groups based on *F*_v_/*F*_m_ ED_50_ thresholds shown in Fig. 1B.

### Confirmation of uptake of Symbiodiniaceae inoculum by coral recruits

**Fig. 2.**
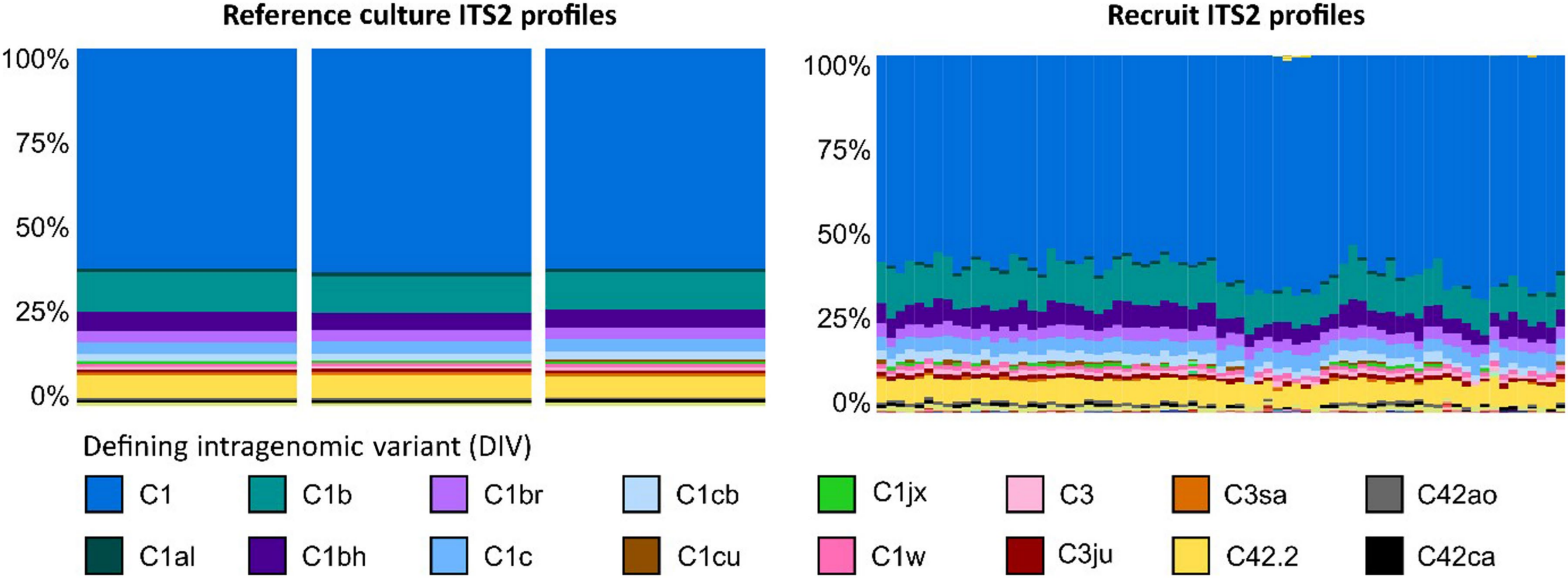
Relative abundance of Symbiodiniaceae ITS2 DIVs. Each bar represents an individual sample, and the colors they are composed of represent the abundance of each sequence defining intragenomic variant (DIV). Reference culture ITS2 profiles for our inoculum were obtained from three pure culture samples of SCF055-01.10 published in (*84*). The recruit ITS2 profiles show recruits given WT (SCF055-01.10) or heat-evolved (SCF055-01.08) Symbiodiniaceae as inoculum (note that there is no difference in ITS2 profile between these two strains; (*43*)].

Recruits were inoculated with either a wild-type (WT10, SCF055-01.10) or HE strain (SS8, SCF055-01.08) of *Cladocopium proliferum*. Internal transcribed spacer 2 (ITS2) metabarcoding was used to confirm that the recruits in the two Symbiodiniaceae treatments consisted of the inoculated strains and that the recruits had not acquired other symbionts from the environment. The *C. proliferum* inoculum was taken up with no major contamination (99.03% ± 0.37 *C. proliferum*). The ITS2 sequences in the profiles of the recruits were present in the reference profile for the HE symbiont and WT symbiont cultures in expected proportions (Fig. 2). Note that variation in the profile data caused by unreliable sequence data was corrected through a supplemental pipeline (text S1). Some minor contamination of other Symbiodiniaceae genera occurred (0.66 ± 0.29% *Durusdinium* and 0.22 ± 0.13% *Fugacium*), likely due to the crustose coralline algae (CCA) used to induce settlement (see Materials and Methods below).

### Performance of recruits under thermal stress

We compared the performance of the various offspring groups inoculated with WT or HE symbionts to assess the impacts of selective breeding and inoculation HE symbionts in isolation and in tandem. The recruits were subjected to ambient (28°C) or elevated (32°C) temperature conditions over 61 days. Degree heating week (DHW) is a measure of heat stress experienced by a coral that accumulates relative to the MMM of its reef (*47*). The offspring of broodstock from Davies Reef (MMM = 28.4°C) accumulated ~3.6 DHW per week and the offspring of broodstock from Moore Reef (MMM = 28.7°C) accumulated ~3.3 DHW per week in the elevated treatment (fig. S3). Bayesian hierarchical modeling was used to assess the performance of the experimental groups, which were compared based on the proportion of the contrast posterior that exceeded zero (for positive effects) or were less than zero (for negative effects), hereafter exceedance probability [*P*_E_; (*48*)]. We considered strong evidence to be *P*_E_ ≥ 0.95, evidence to be 0.95 < *P*_E_ ≥ 0.9, weak evidence to be 0.9 > *P*_E_ ≥ 0.85, and no evidence to be <0.85.

#### Effects of selective breeding on recruit performance

**Fig. 3.**
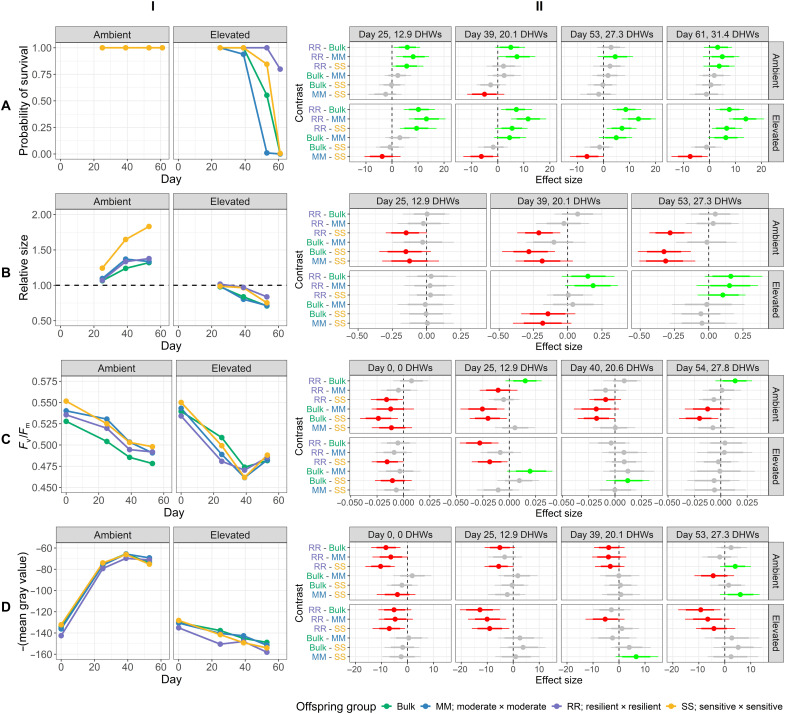
Performance of the Davies Reef offspring of heat resilient (RR; purple), heat sensitive (SS; yellow), and moderately heat resilient (MM; blue) broodstock, and the offspring of colonies crossed in bulk (Bulk; green). The recruits were inoculated with WT *C. proliferum* and subjected to 61 days of ambient (28°C) and elevated (32°C) temperature conditions. Panels (**A**) to (**D**) depict the survival (probability of surviving), growth, photochemical efficiency (*F*_v_/*F*_m_), and color of the recruits, respectively. The first column (I) depicts the estimated marginal means of the posterior distributions from Bayesian models for each performance metric over time. Growth is shown as size at a given time point divided by size at day 0; a dashed line at a relative size of one delineates growing and shrinking coral. Color is displayed as the inverse additive of mean grey value such that higher values indicate darker recruits. The second column (II) shows comparisons among the Davies Reef offspring groups over time, displaying the 80% (thin line) and 95% (thick line) highest posterior density (HPD) intervals of the log(odds ratios) for survivorship and growth data and of the difference in estimated marginal means for *F*_v_/*F*_m_ and color data of pairs of offspring groups (effect size) in the same temperature treatment and at the same time point [number of days and degree heating weeks (DHWs) accrued are listed for each time point] from the posterior distributions of Bayesian-mixed effects modeling. Green intervals indicate that 85% HPD of the effect size exceeds zero, meaning the first offspring group had a higher value for the trait compared to the second. Red intervals indicate that 85% HPD of the effect size is less than zero, meaning the first offspring group had lower values for the trait compared to the second.

**Fig. 4.**
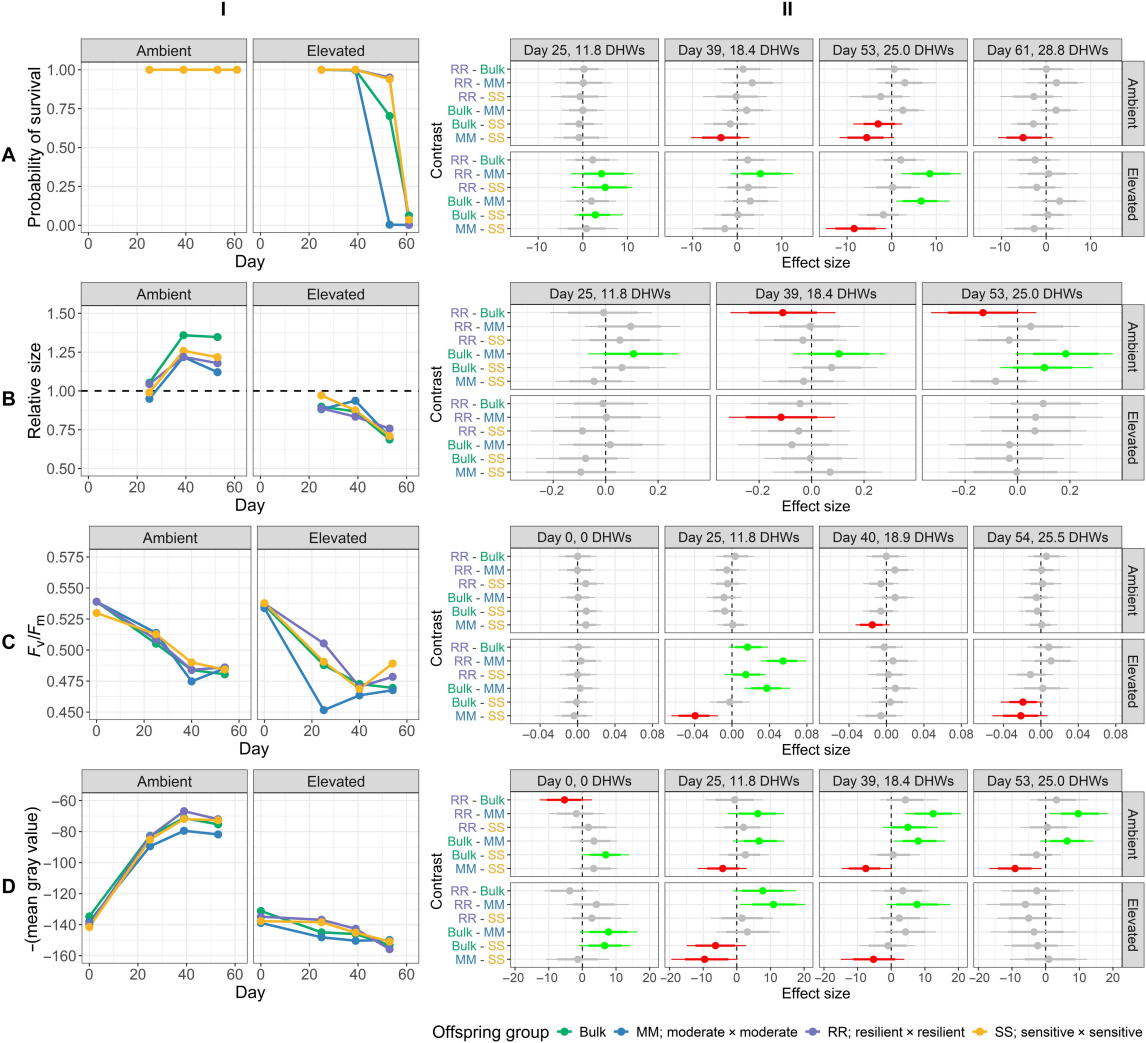
Performance of the Moore Reef offspring of heat resilient (RR; purple), heat sensitive (SS; yellow), moderately heat resilient (MM; blue) broodstock, and the offspring of colonies crossed in bulk (Bulk; green). The recruits were inoculated with WT *C. proliferum* and subjected to 61 days of ambient (28°C) and elevated (32°C) temperature conditions. Panels (**A**) to (**D**) depict the survival (probability of surviving), growth, photochemical efficiency (*F*_v_/*F*_m_), and color of the recruits, respectively. The first column (I) depicts the estimated marginal means of the posterior distributions from Bayesian models for each performance metric over time. Growth is shown as size at a given time point divided by size at day 0; a dashed line at a relative size of one delineates growing and shrinking coral. Color is displayed as the inverse additive of mean grey value such that higher values indicate darker recruits. The second column (II) shows comparisons among the Moore Reef offspring groups over time, displaying the 80% (thin line) and 95% (thick line) HPD intervals of the log(odds ratios) for survivorship and growth data and of the difference in estimated marginal means for *F*_v_/*F*_m_ and color data of pairs of offspring groups (effect size) in the same temperature treatment and at the same time point (number of days and DHWs accrued are listed for each time point) from the posterior distributions of Bayesian mixed effects modeling. Green intervals indicate that 85% HPD of the effect size exceeds zero, meaning the first offspring group had a higher value for the trait compared to the second. Red intervals indicate that 85% HPD of the effect size is less than zero, meaning the first offspring group had lower values for the trait compared to the second.

Selective breeding had broodstock-dependent effects on the performance of the recruits with WT symbionts under ambient and elevated conditions (Figs. 3 and 4). Overall (specifics detailed below), offspring of the most heat-tolerant Davies Reef broodstock (RR) displayed enhanced survival (Fig. 3A) and growth (Fig. 3B) under elevated conditions but simultaneously some evidence of increased bleaching susceptibility (*F*_v_/*F*_m_ and color; Fig. 3, C and D) under elevated conditions and limited (relative only to the offspring of the most heat-sensitive broodstock; SS) evidence of reduced growth (Fig. 3B) under ambient conditions. In contrast, Moore Reef RR recruits displayed evidence of mild bleaching resilience (*F*_v_/*F*_m_ and color; Fig. 4, C and D) and some evidence of enhanced survival (relative to two of the three control offspring groups; Fig. 4A) under elevated conditions and reduced growth (Fig. 4B) under ambient (relative to offspring produced in bulk, irrespective of broodstock heat tolerance; Bulk) and elevated (relative to the offspring of moderately heat-tolerant broodstock; MM) conditions.

Survival was high across the offspring groups under ambient conditions (Figs. 3A and 4A). However, the few mortalities that occurred among the offspring groups indicated that there was weak to strong evidence (*P*_E_ ≥ 0.881) that Davies Reef RR had enhanced survival under ambient conditions relative to Davies Reef Bulk, MM, and SS at 25 and 61 days, Bulk and MM at 39, and MM at 53 days (Fig. 3A). Moore Reef RR had similar survival to Moore Reef Bulk, MM, and SS at each time point under ambient conditions (*P*_E_ < 0.850; Fig. 4A). Under elevated conditions and with respect to Davies Reef recruits, there was strong evidence that RR had enhanced survival relative to bulk, MM, and SS at 12.9, 20.1, 27.3, and 31.4 DHW (*P*_E_ ≥ 0.956; Fig. 3A). With respect to Moore Reef recruits under elevated conditions, there was weak to strong evidence (*P*_E_ ≥ 0.875) that RR had enhanced survival relative to MM at 11.8, 18.4, and 25.0 DHW and evidence that RR had enhanced survival relative to SS at 11.8 DHW (*P*_E_ = 0.928), but no evidence that RR had enhanced survival relative to Bulk (*P*_E_ < 0.850; Fig. 4A).

Regarding the Davies Reef recruits, there was strong evidence that RR had grown less than SS (but not Bulk or MM) at 25, 39, and 53 days (*P*_E_ ≥ 0.968) under ambient conditions (Fig. 3B). Further, there was weak to strong evidence (*P*_E_ ≥ 0.880) that Davies Reef RR had grown more than Davies Reef Bulk and MM at 20.1 and 27.3 DHW and SS at 27.3 DHW (Fig. 3B). Meanwhile and under ambient conditions, Moore Reef RR displayed similar growth to Moore Reef SS and MM (*P*_E_ < 0.850) but reduced growth relative to Moore Reef Bulk at days 39 (*P*_E_ = 0.863) and 53 (*P*_E_ = 0.900; Fig. 4B). Moore Reef RR had reduced growth relative to Moore Reef MM at 18.4 DHW (*P*_E_ = 0.859). Otherwise, the growth of the Moore Reef offspring groups was similar under elevated conditions (*P*_E_ < 0.850; Fig. 4B).

Davies Reef RR displayed weak evidence to evidence (*P*_E_ ≥ 0.877) of both enhanced (relative to Davies Reef Bulk) and reduced (relative to Davies Reef MM and SS) *F*_v_/*F*_m_ under ambient conditions and strong evidence (*P*_E_ ≥ 0.988) of reduced *F*_v_/*F*_m_ relative to Davies Reef Bulk and SS at 12.9 DHW (Fig. 3C). Moore Reef RR had similar *F*_v_/*F*_m_ to Moore Reef Bulk, MM, and SS at each time point under ambient conditions (*P*_E_ < 0.850; Fig. 4C). Meanwhile, there was weak to strong evidence (*P*_E_ ≥ 0.897) that Moore Reef RR had enhanced *F*_v_/*F*_m_ relative to Bulk, MM, and SS at 11.8 DHW (Fig. 4C). It must be noted that *F*_v_/*F*_m_ values under elevated temperatures were higher than expected in bleached adult coral (*43*) and declined in both ambient and elevated treatments, suggesting that recruit *F*_v_/*F*_m_ responses reflected experimental conditions beyond temperature stress.

There was weak to strong evidence that Davies Reef RR were lighter than each of the Davies Reef control groups at two or more time points under ambient and elevated conditions (*P*_E_ ≥ 0.854; Fig. 3D). However, there was weak evidence that Davies Reef RR were darker than Davies Reef SS at day 53 under ambient conditions (*P*_E_ = 0.894; Fig. 3D). For the Moore Reef recruits under ambient conditions beyond day 0, RR was similar in color to Bulk (*P*_E_ < 0.850), but there was weak to strong evidence (*P*_E_ ≥ 0.893) that RR was darker than MM at 25, 39, and 53 days and SS at 39 days (Fig. 4D). Under elevated conditions, there was evidence to strong evidence (*P*_E_ ≥ 0.942) that Moore Reef RR were darker than Bulk and MM (but not SS) at 11.8 and MM at 18.4 DHW (Fig. 4D).

#### *Effects of inoculation with heat-evolved* Symbiodiniaceae *on recruit performance*

**Fig. 5.**
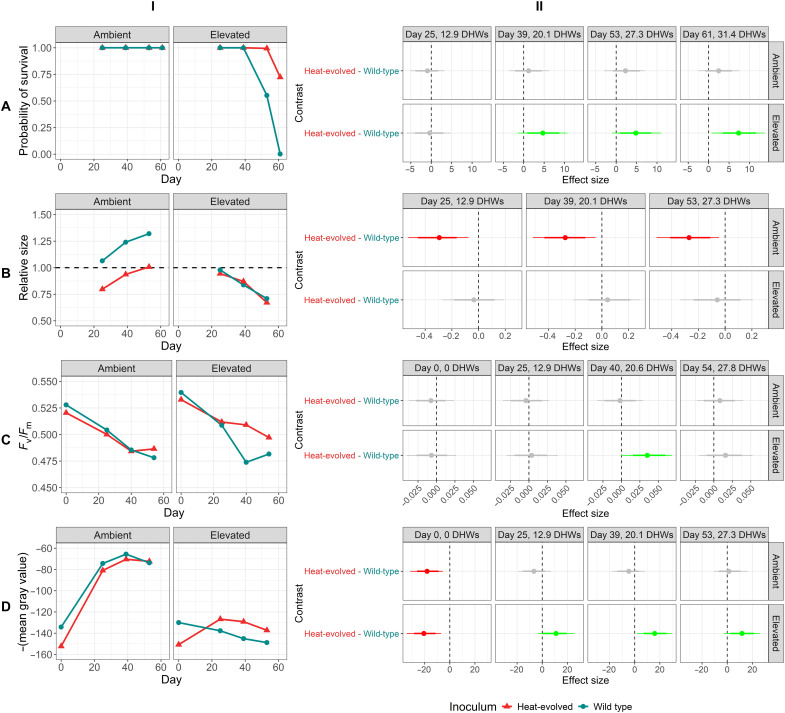
Performance of Davies Reef Bulk offspring inoculated with WT (blue circles) and heat-evolved (red triangles) *C. proliferum* under 61 days of ambient (28°C) and elevated (32°C) temperature conditions. Panels (**A**) to (**D**) depict the survival (probability of surviving), growth, photochemical efficiency (*F*_v_/*F*_m_), and color of the recruits, respectively. The first column (I) depicts the estimated marginal means of the posterior distributions from Bayesian models for each performance metric over time. Growth is shown as size at a given time point divided by size at day 0; a dashed line at a relative size of one delineates growing and shrinking coral. Color is displayed as the inverse additive of mean gray value, such that higher values indicate darker recruits. The second column (II) shows comparisons between the Davies Reef Bulk offspring inoculated with WT and HE over time, displaying the 80% (thin line) and 95% (thick line) HPD intervals of the log(odds ratios) for survival and growth data and of the difference in estimated marginal means for *F*_v_/*F*_m_ and color data of the two Symbiodiniaceae treatments (effect size) in the same temperature treatment and at the same time point (number of days and DHWs accrued are listed for each time point) from the posterior distributions of Bayesian-mixed effects modeling. Green intervals indicate that 85% HPD of the effect size exceeds zero, meaning recruits inoculated with HE had a higher value for the trait compared to those inoculated with WT. Red intervals indicate that 85% HPD of the effect size is less than zero, meaning recruits inoculated with HE had a lower value for the trait compared to those inoculated with WT.

**Fig. 6.**
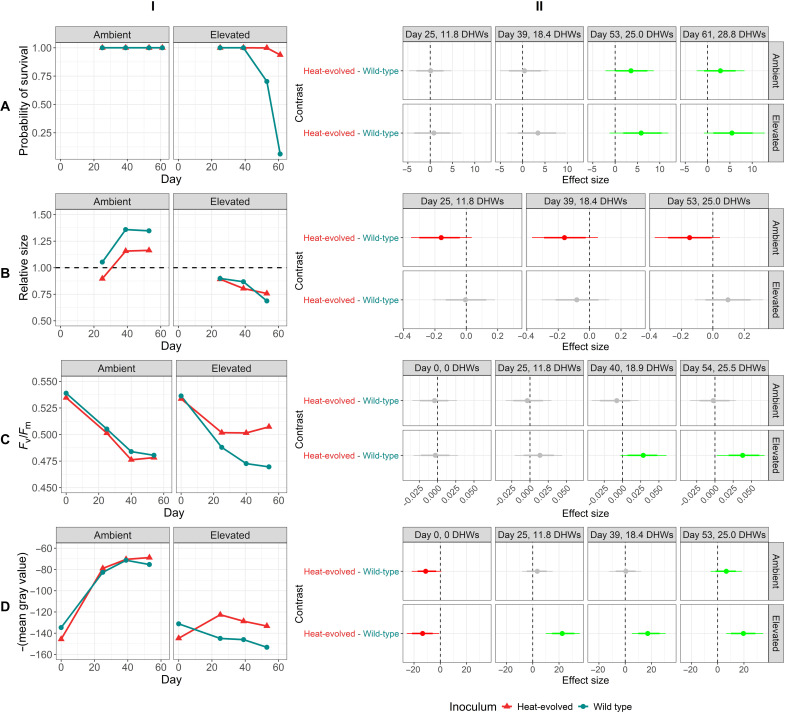
Performance of Moore Reef Bulk offspring inoculated with WT (blue circles) and heat-evolved (red triangles) *C. proliferum* under 61 days of ambient (28°C) and elevated (32°C) temperature conditions. Panels (**A**) to (**D**) depict the survival (probability of surviving), growth, photochemical efficiency (*F*_v_/*F*_m_), and color of the recruits, respectively. The first column (I) depicts the estimated marginal means of the posterior distributions from Bayesian models for each performance metric over time. Growth is shown as size at a given time point divided by size at day 0; a dashed line at a relative size of one delineates growing and shrinking coral. Color is displayed as the inverse additive of mean grey value such that higher values indicate darker recruits. The second column (II) shows comparisons between the Moore Reef Bulk offspring inoculated with WT and HE over time, displaying the 80% (thin line) and 95% (thick line) HPD intervals of the log(odds ratios) for survival and growth data and of the difference in estimated marginal means for *F*_v_/*F*_m_ and color data of the two Symbiodiniaceae treatments (effect size) in the same temperature treatment and at the same time point (number of days and DHWs accrued are listed for each time point) from the posterior distributions of Bayesian-mixed effects modeling. Green intervals indicate that 85% HPD of the effect size exceeds zero, meaning recruits inoculated with HE had a higher value for the trait compared to those inoculated with WT. Red intervals indicate that 85% HPD of the effect size is less than zero, meaning recruits inoculated with HE had a lower value for the trait compared to those inoculated with WT.

We compared the performance of Bulk recruits inoculated with HE symbionts and WT symbionts to assess the effect of inoculation with HE symbionts as an assisted evolution intervention. Recruits with HE symbionts displayed enhanced survival (Figs. 5A and 6A) and evidence of enhanced bleaching resilience (*F*_v_/*F*_m_ and color; Figs. 5 and 6, C and D) under elevated temperature with a trade-off of reduced growth (Figs. 5B and 6B) under ambient conditions.

Survival was high across the Symbiodiniaceae treatments under ambient conditions (Figs. 5A and 6A); however, the few mortalities that occurred indicated that there was weak evidence (*P*_E_ ≥ 0.860) that inoculation with HE symbionts enhanced the survival of Moore Reef recruits at 53 and 61 days (Fig. 6A). Under elevated conditions, there was evidence to strong evidence (*P*_E_ ≥ 0.940) that inoculation with HE symbionts enhanced the survival of Davies Reef recruits at 20.1, 27.3, and 31.4 DHW and Moore Reef recruits at 25.0 and 28.8 DHW (Figs. 5A and 6A).

There was evidence to strong evidence (*P*_E_ ≥ 0.915) that inoculation with HE symbionts reduced Davies Reef and Moore Reef recruit growth under ambient conditions at days 25, 39, and 53 (Figs. 5B and 6B). Starting sizes of recruits in each Symbiodiniaceae treatment group were not significantly different (difference in mean size = 0.02 mm^2^; *F* = 0.41 and *P* = 0.523). Therefore, these differences in proportional growth under ambient conditions likely reflect differences in net growth over the first 25 days of the experiment (Figs. 5B and 6B). However, under elevated conditions, the negative growths of Davies and Moore Reef Bulk were similar when they were inoculated with WT symbionts or HE symbionts (*P*_E_ < 0.850; Figs. 5B and 6B).

There was strong evidence (*P*_E_ ≥ 0.961) that inoculation with HE symbionts enhanced *F*_v_/*F*_m_ of Davies Reef Bulk at 20.6 DHW and Moore Reef Bulk at 18.9 and 25.5 DHW (Figs. 5C and 6C). Otherwise, the *F*_v_/*F*_m_ of Bulk recruits inoculated with HE symbionts and WT symbionts was comparable under ambient and elevated conditions (*P*_E_ < 0.850; Figs. 5C and 6C).

Bulk from Davies Reef or Moore Reef inoculated with HE symbionts was lighter than those inoculated with WT symbionts at the beginning of the experiment (*P*_E_ ≥ 973; Figs. 5D and 6D). However, at 25, 39, and 53 days, the color of Bulk from Davies Reef and Moore Reef inoculated with HE symbionts and WT symbionts was similar (*P*_E_ < 0.850) in the ambient treatment (Figs. 5D and 6D) with one exception: There was weak evidence that Moore Reef recruits were darker when inoculated with HE symbionts compared to WT symbionts at 53 days under ambient conditions (*P*_E_ = 0.859). Alternatively, there was evidence to strong evidence (*P*_E_ ≥ 0.933) that Davies Reef Bulk inoculated with HE symbionts were darker than those inoculated with WT symbionts at 12.9, 20.1, and 27.3 DHW (Fig. 5D) and that Moore Reef Bulk inoculated with HE symbionts were darker than those inoculated with WT symbionts at 11.8, 18.4, and 25 DHW (Fig. 6D).

#### Effect of combining interventions on recruit performance

**Fig. 7.**
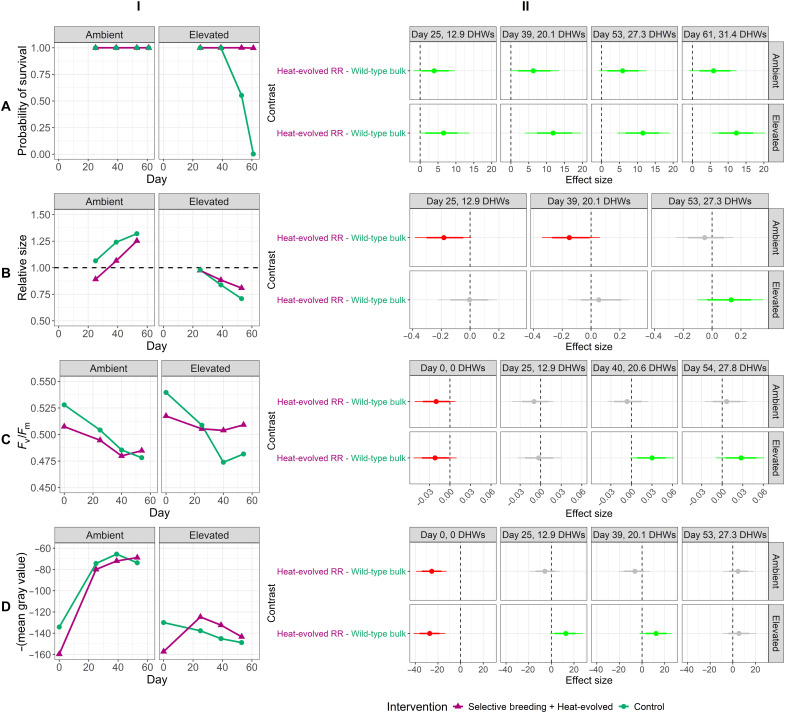
Performance of Davies Reef recruits from the control (Bulk inoculated with WT *C. proliferum*; green circles) and combined selective breeding and inoculation with HE symbionts intervention (RR recruits inoculated with heat-evolved *C. proliferum*; pink triangles) experimental groups under 61 days of ambient (28°C) and elevated (32°C) temperature conditions. Panels (**A**) to (**D**) depict recruit survival (probability of surviving), growth, photochemical efficiency (*F*_v_/*F*_m_), and color, respectively. The first column (I) depicts the estimated marginal means of the posterior distributions from Bayesian models for each performance metric over time. Growth of the recruits is shown as size at a given time point divided by size at day 0; a dashed line at a relative size of one delineates growing and shrinking coral. Color is displayed as the inverse additive of mean grey value such that higher values indicate darker recruits. The second column (II) shows comparisons between the control and combined intervention Davies Reef experimental groups over time, displaying the 80% (thin line) and 95% (thick line) HPD intervals of the log(odds ratios) for survival and growth data and differences in estimated marginal means for *F*_v_/*F*_m_ and color data of the two experimental groups in the same temperature treatment and at the same time point (number of days and DHWs accrued are listed for each time point) from the posterior distributions of Bayesian mixed effects modeling. Green intervals indicate that 85% HPD of the effect size exceeds zero, meaning recruits from the combined intervention group had higher values for the trait compared to those from the control group. Red intervals indicate that 85% HPD of the effect size is less than zero, meaning recruits from the combined intervention group had lower values for the trait compared to those from the control group.

**Fig. 8.**
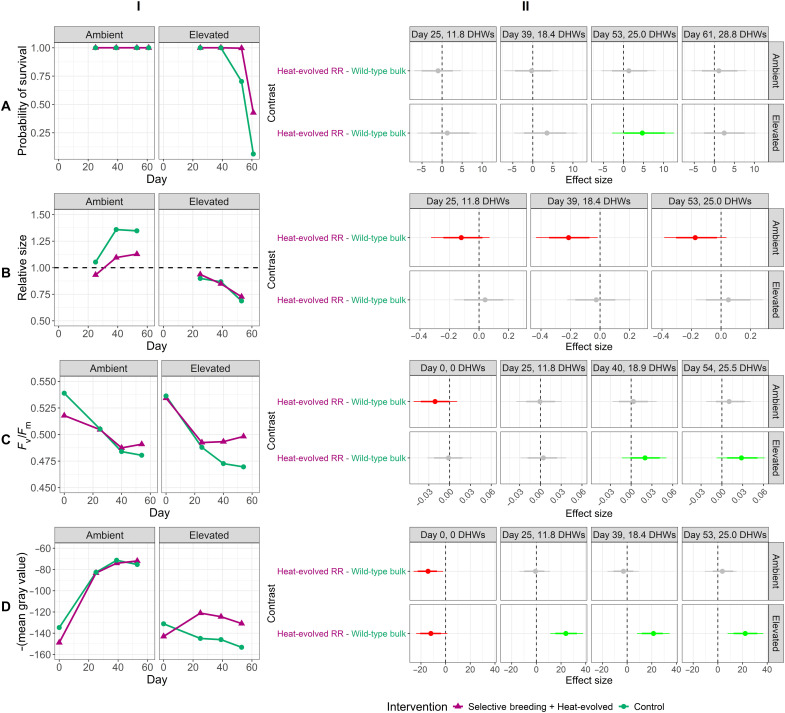
Performance of Moore Reef recruits from the control (Bulk inoculated with WT *C. proliferum*; green circles) and combined selective breeding and inoculation with HE symbionts intervention (RR recruits inoculated with heat-evolved *C. proliferum*; pink triangles) experimental groups under 61 days of ambient (28°C) and elevated (32°C) temperature conditions. Panels (**A**), (**B**), (**C**), and (**D**) depict recruit survival (probability of surviving), growth, photochemical efficiency (*F*_v_/*F*_m_), and color, respectively. The first column (I) depicts the estimated marginal means of the posterior distributions from Bayesian models for each performance metric over time. Growth of the recruits is shown as size at a given time point divided by size at day 0; a dashed line at a relative size of one delineates growing and shrinking coral. Color is displayed as the inverse additive of mean grey value such that higher values indicate darker recruits. The second column (II) shows comparisons between the control and combined intervention Moore Reef experimental groups over time, displaying the 80% (thin line) and 95% (thick line) HPD intervals of the log(odds ratios) for survival and growth data and differences in estimated marginal means for *F*_v_/*F*_m_ and color data of the two experimental groups in the same temperature treatment and at the same time point (number of days and DHWs accrued are listed for each time point) from the posterior distributions of Bayesian mixed effects modeling. Green intervals indicate that 85% HPD of the effect size exceeds zero, meaning recruits from the combined intervention group had higher values for the trait compared to those from the control group. Red intervals indicate that 85% HPD of the effect size is less than zero, meaning recruits from the combined intervention group had lower values for the trait compared to those from the control group.

Comparison of the performance of Bulk inoculated with WT symbionts and RR inoculated with HE symbionts to test the effect of selective breeding and inoculation with HE symbionts in combination, showed evidence of enhancement in survival and bleaching resilience (color and *F*_v_/*F*_m_), but a trade-off against growth was observed under ambient conditions (Figs. 7 and 8).

Survival was high across the control and combined intervention treatments under ambient conditions (Figs. 7A and 8A); however, the few mortalities that occurred indicated that there was evidence to strong evidence (*P*_E_ ≥ 0.906) that combining interventions enhanced Davies Reef recruit survival at days 25, 39, 53, and 61 (Fig. 7A). Under elevated conditions, there was strong evidence (*P*_E_ ≥ 0.973) that combining interventions enhanced Davies Reef recruit survival at 12.9, 20.1, 27.3, and 31.4 DHW (Fig. 7A). However, combining interventions did not enhance or reduce (*P*_E_ < 0.850) Moore Reef recruit survival under ambient or elevated conditions with one exception: There was weak evidence that combining interventions enhanced recruit survival at 25.0 DHW (*P*_E_ = 0.888; Fig. 8A).

There was weak to strong evidence (*P*_E_ ≥ 0.879) that combining interventions reduced Davies Reef recruit growth at 25 and 39 days and Moore Reef recruit growth at 25, 39, and 53 days under ambient conditions (Figs. 7B and 8B). Conversely, there was weak evidence that combining interventions enhanced Davies Reef recruit growth at 27.3 DHW (*P*_E_ = 0.865; Fig. 8B). Combining interventions did not influence Moore Reef recruit growth under elevated conditions (*P*_E_ < 0.850; Fig. 8B).

There was weak to strong evidence (*P*_E_ ≥ 0.888) that combining interventions enhanced Davies Reef recruit *F*_v_/*F*_m_ at 20.6 and 27.8 DHW (Fig. 7C) and Moore Reef recruit *F*_v_/*F*_m_ at 18.9 and 25.5 DHW (Fig. 8C). Combining interventions did not influence recruit *F*_v_/*F*_m_ under ambient temperatures beyond day 0 when there was no to moderate evidence (*P*_E_ ≤ 0.915) that recruits from the combined intervention treatment had reduced *F*_v_/*F*_m_ (Figs. 7C and 8C).

Davies Reef recruits from the combined intervention treatment were lighter than Bulk inoculated with WT symbionts at the beginning of the experiment (*P*_E_ ≥ 0.999), but there was strong evidence (*P*_E_ ≥ 0.955) that they were darker than Bulk inoculated with WT symbionts at 12.9 and 20.1 DHW (Fig. 7D). Similarly, there was strong evidence (*P*_E_ ≥ 0.961) that Moore Reef recruits from the combined intervention treatment were lighter than Bulk inoculated with WT symbionts at the beginning of the experiment but darker than Bulk inoculated with WT symbionts at 11.8, 18.4, and 25.0 DHW (Fig. 8D). Combining interventions otherwise did not affect the color of the Moore Reef or Davies Reef recruits under ambient conditions (*P*_E_ < 0.850; Figs. 7D and 8D).

#### *Comparing the effects of selective breeding and inoculation with heat-evolved* Symbiodiniaceae *in isolation and tandem on recruit performance*

**Fig. 9.**
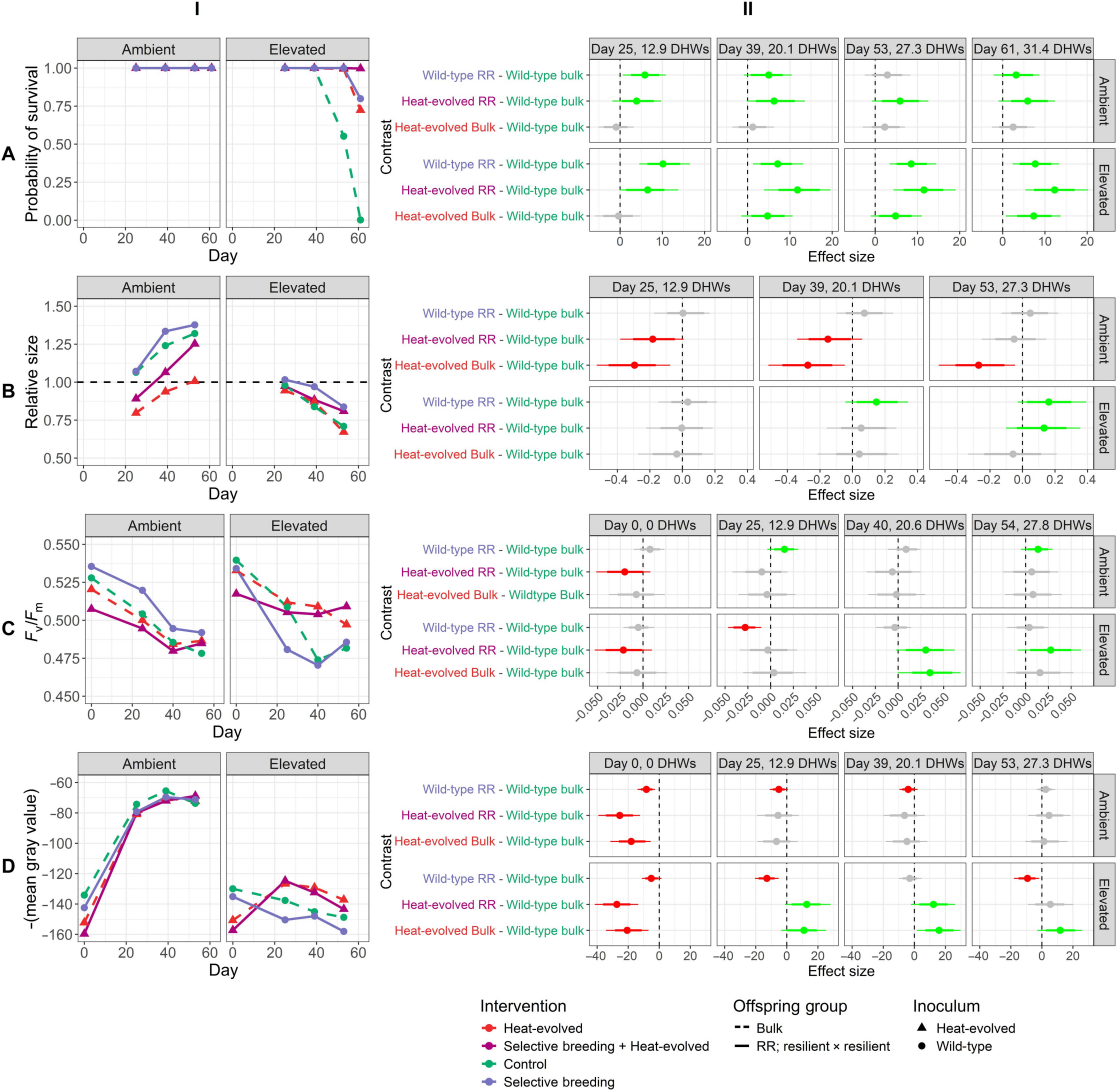
Performance of Davies Reef recruits from each intervention treatment under 61 days of ambient (28°C) and elevated (32°C) temperature conditions: inoculation with heat-evolved Symbiodiniaceae (red), selective breeding (purple), both interventions combined (pink), and control (green). Recruits inoculated with heat-evolved and WT *C. proliferum* are shown as triangles and circles, respectively. RR and Bulk recruits are shown as continuous and broken lines, respectively. Panels (**A**), (**B**), (**C**), and (**D**) depict recruit survival (probability of surviving), growth, photochemical efficiency (Fv/Fm), and color, respectively. Column I depicts the estimated marginal means of the posterior distributions from Bayesian models for each performance metric over time. Growth is shown as size at a given time point divided by size at day 0; a dashed line at a relative size of one delineates growing and shrinking coral. Color is displayed as the inverse additive of mean gray value, such that higher values indicate darker recruits. Column II shows comparisons between the Davies Reef control and intervention experimental groups over time, displaying the 80% (thin line) and 95% (thick line) HPD intervals of the log(odds ratios) for survival and growth data and differences in estimated marginal means for *F*_v_/*F*_m_ and color data between the two groups in the same temperature treatment and at the same time point (number of days and DHWs are listed for each time point) from the posterior distributions of Bayesian-mixed effects modeling. Green intervals indicate that 85% HPD of the effect size exceeds zero, meaning recruits from the intervention group had higher values for the trait compared to those from the control group. Red intervals indicate that 85% HPD of the effect size is less than zero, meaning recruits from the intervention group had lower values for the trait compared to those from the control group.

**Fig. 10.**
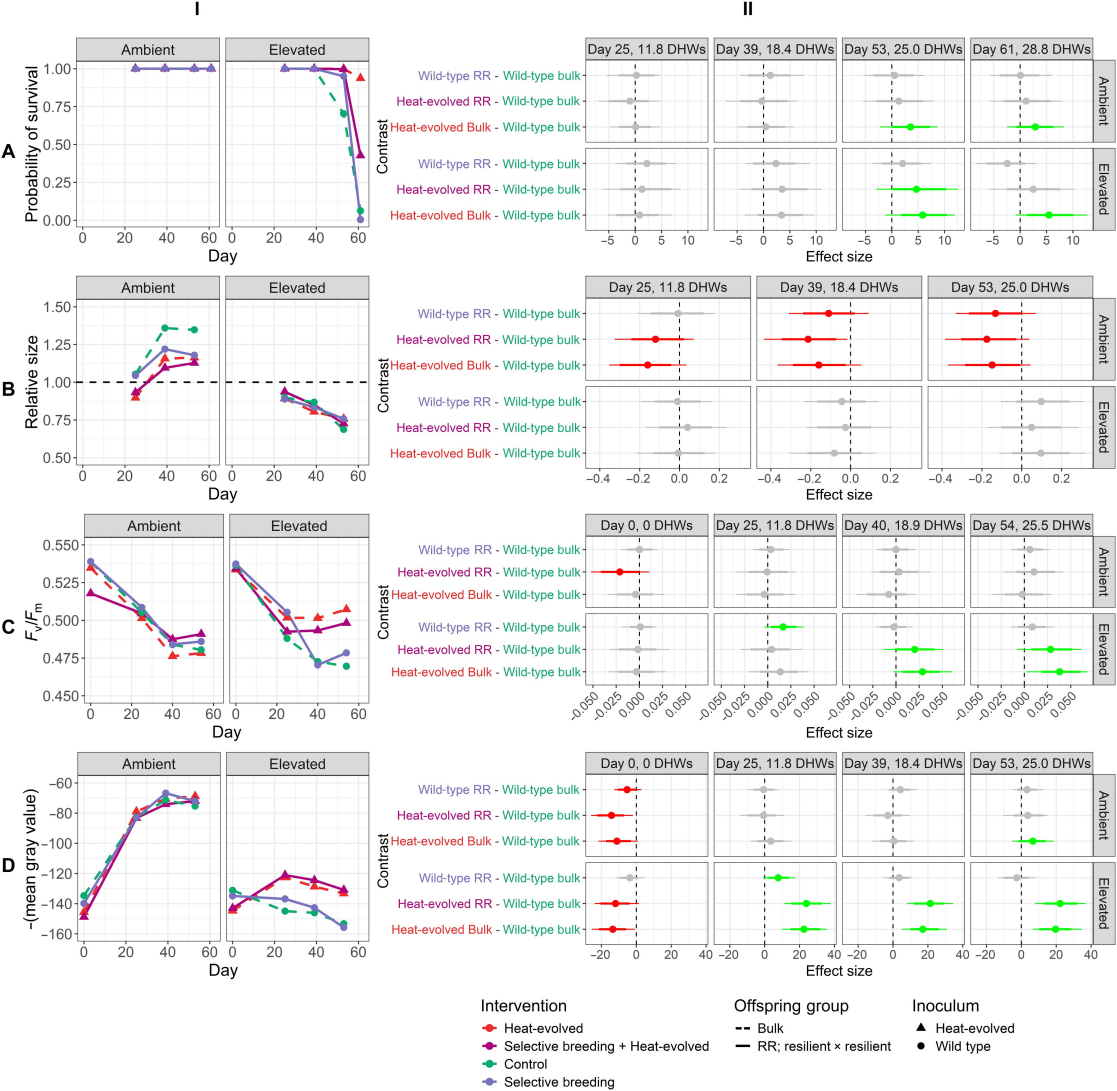
Performance of Moore Reef recruits from each intervention treatment under 61 days of ambient (28°C) and elevated (32°C) temperature conditions: inoculation with heat-evolved Symbiodiniaceae (red), selective breeding (purple), both interventions combined (pink), and control (green). Recruits inoculated with heat-evolved and WT *C. proliferum* are shown as triangles and circles, respectively. RR and Bulk recruits are shown as continuous and broken lines, respectively. Panels (**A**), (**B**), (**C**), and (**D**) depict recruit survival (probability of surviving), growth, photochemical efficiency (Fv/Fm), and color, respectively. Column I depicts the estimated marginal means of the posterior distributions from Bayesian models for each performance metric over time. Growth is shown as size at a given time point divided by size at day 0; a dashed line at a relative size of one delineates growing and shrinking coral. Color is displayed as the inverse additive of mean grey value, such that higher values indicate darker recruits. Column II shows comparisons between the Moore Reef control and intervention experimental groups over time, displaying the 80% (thin line) and 95% (thick line) HPD intervals of the log(odds ratios) for survival and growth data and differences in estimated marginal means for *F*_v_/*F*_m_ and color data between the two groups in the same temperature treatment and at the same time point (number of days and DHWs are listed for each time point) from the posterior distributions of Bayesian mixed effects modeling. Green intervals indicate that 85% HPD of the effect size exceeds zero, meaning recruits from the intervention group had higher values for the trait compared to those from the control group. Red intervals indicate that 85% HPD of the effect size is less than zero, meaning recruits from the intervention group had lower values for the trait compared to those from the control group.

We compared the relative performance of three intervention groups—RR inoculated with WT, Bulk inoculated with HE, and RR inoculated with HE—each benchmarked against the control group (Bulk inoculated with WT symbionts)—to compare the effects of selective breeding, HE inoculation, and their combination (Figs. 9 and 10). The following report of the reductions and enhancements in recruit performance due to the various interventions are supported by *P*_E_ ≥ 0.850.

With respect to the Davies Reef recruits, combining interventions enhanced survival at all time points under ambient and elevated conditions, selective breeding enhanced survival at all time points under elevated conditions and all but one time point under ambient conditions, while inoculation with HE symbionts enhanced survival at all but one time point under elevated conditions but not under ambient conditions (Fig. 9A). Further, while inoculation with HE symbionts enhanced survival at 53 and 61 days under ambient conditions and 25.0 and 28.8 DHW, combining interventions only enhanced survival at 25.0 DHW, and selective breeding did not enhance survival in the Moore Reef recruits (Fig. 10A).

Inoculation with HE symbionts (25, 39, and 53 days) and combining interventions (25 and 39 days) reduced growth in the Davies Reef recruits under ambient conditions, while selective breeding did not (Fig. 9B). Also, in the Davies Reef recruits, selective breeding enhanced growth at 20.1 and 27.3 DHW, and combining interventions enhanced growth at 27.3 DHW, while inoculation with HE symbionts did not enhance growth under elevated conditions (Fig. 9B). In the Moore Reef recruits, the interventions did not influence recruit growth under elevated conditions, but inoculation with HE symbionts and combining interventions reduced growth at 25, 39, and 53 days, and selective breeding reduced growth at 39 and 53 days under ambient conditions (Fig. 10B).

In the Davies Reef recruits, selective breeding enhanced *F*_v_/*F*_m_ under ambient conditions (days 25 and 54), while inoculation with HE symbionts and combining interventions did not (Fig. 9C). In the Davies Reef recruits grown under elevated conditions, inoculation with HE symbionts enhanced *F*_v_/*F*_m_ at 20.6 DHW, and combining interventions enhanced *F*_v_/*F*_m_ at 20.6 DHW and 27.8 DHW, while selective breeding did not enhance *F*_v_/*F*_m_ but reduced it at 12.9 DHW (Fig. 9C). In the Moore Reef recruits, the interventions did not influence *F*_v_/*F*_m_ under ambient conditions with the exception that inoculation with HE symbionts reduced *F*_v_/*F*_m_ at day 0 (Fig. 10C). Also, in the Moore Reef recruits, inoculation with HE symbionts and combining interventions enhanced *F*_v_/*F*_m_ at 18.9 and 25.5 DHW, and selective breeding enhanced *F*_v_/*F*_m_ at 11.8 DHW (Fig. 10C).

All the interventions resulted in the recruits being lighter at day 0. In the Davies Reef recruits, selective breeding resulted in the recruits being lighter at 25 and 39 days under ambient conditions and 12.9 and 27.3 DHW (Fig. 9D). Inoculation with HE symbionts and combining interventions did not affect Davies Reef recruit color beyond day 0 of the experiment under ambient conditions but resulted in them being darker at 12.9, 20.1, and 27.3 DHW and 12.9 and 20.1 DHW, respectively (Fig. 9D). In the Moore Reef recruits, inoculation with HE symbionts resulted in the recruits being darker at day 53, but, otherwise, the interventions did not affect recruit color beyond day 0 of the experiment under ambient conditions (Fig. 10D). In the Moore Reef recruits under elevated conditions, selective breeding resulted in the recruits being darker at 11.8 DHW, while inoculation with HE symbionts and combining interventions resulted in them being darker at 11.8, 18.4, and 25.0 DHW (Fig. 10D).

## DISCUSSION

The Davies and Moore Reef recruits studied here were exposed to levels of heat stress (31.4 DHW and 28.8 DHW, respectively) that may not eventuate on the GBR under more conservative shared socioeconomic pathway (SSP) projections but could be recorded on the GBR under more severe scenarios (*49*). In keeping with previous results (*50*, *51*), the recruits were relatively tolerant to heat stress such that most experimental groups had >90% survival after ~20 DHW; adult and larger juvenile corals on natural reefs are expected to experience >80% mortality after accruing this amount of heat stress [Fig. 3; (*52*)]. The low-light levels that the recruits were exposed to in this experiment may have contributed to their tolerance because thermal bleaching responses are exacerbated under high light (*53*). Nonetheless, the levels of light within this experiment are ecologically relevant and comparable to the cryptic locations on reefs that recruits typically settle at (*54*, *55*). Further, the patterns in recruit color, survival, and growth are roughly similar to observations reported in other temperature stress experiments on *Acropora* recruits (*36*, *56*–*58*). Our experiments on these coral recruits showed selective breeding based on broodstock phenotypes, and inoculation with HE used in isolation and in tandem has significant effects on the performance of recruits under ambient and elevated temperatures, which will be discussed below.

### Selective breeding based on broodstock phenotypes has variable outcomes

Several approaches have been trialed to enhance coral heat tolerance through selective breeding based on broodstock heat tolerance phenotypes, with some promising outcomes. Drury *et al.* (*31*) used *Montipora capitata* bleaching phenotypes under a natural heat wave to inform selective breeding, where selection may have targeted either host genetics or symbiont communities, which are inherited through vertical transmission in this species. In the larval and recruit life stages, offspring of bleaching-resilient broodstock had enhanced survival compared to offspring of bleaching sensitive parents after 8 days and 1 month of exposure to elevated temperatures, respectively. However, compared to a bulk cross (analogous to the Bulk offspring group used here), enhanced survival in offspring of bleaching-resilient parents was evident only at the recruit stage, not in the larvae. Humanes *et al.* (*23*) assessed survivorship under rapid (1 week) and moderate (1 month) heat stress assays to identify *Acropora digitifera* parental broodstock with high and low heat tolerance. When used to inform breeding, selecting broodstock based on the rapid heat stress assay enhanced survival of adult offspring under rapid stress conditions only and did not enhance survival under moderate heat stress (*23*). However, selecting broodstock based on the moderate heat stress assay did boost offspring survival under moderate heat stress conditions (*23*). Here, we assessed the efficacy of selective breeding informed by *F*_v_/*F*_m_ measured under a rapid heat stress assay (8 hours) on enhancing survival, growth, and bleaching resilience (*F*_v_/*F*_m_ and color) of *A. spathulata* recruit offspring under a 2-month stress test and found variable outcomes across traits. Furthermore, the selective breeding of the Moore and Davies Reef broodstock had different outcomes with respect to recruit heat tolerance. Selective breeding of the most heat-tolerant Davies Reef broodstock resulted in enhanced survival (relative to all control groups at all time points during stress testing) and growth (relative to all control groups at one or more time points during stress testing), and we found some evidence of increased bleaching susceptibility (*F*_v_/*F*_m_ and color) under elevated conditions. In contrast, Moore Reef RR recruits did not show enhanced survival or growth relative to all control groups but did display evidence of mild bleaching resilience (*F*_v_/*F*_m_ and color) under elevated conditions. Variation in the success of selective breeding among broodstock subpopulations/populations and approaches is not unexpected and can be explained by genetic, maternal, and environmental determinants of trait variation.

Coral heat tolerance is dependent on the type of thermal stress experienced and the metric used to assess heat tolerance (*23*). This study demonstrates that selective breeding based on broodstock *F*_v_/*F*_m_ under a rapid heat stress assay can enhance recruit heat tolerance under conditions of extended heat stress. However, the selective breeding method used here did not yield consistent outcomes between the Moore and Davies Reef offspring, nor did it reliably produce the expected results. For example, the color of Moore Reef RR indicated that they were more bleaching resilient than Bulk and/or MM recruits but not compared to offspring of the most thermally sensitive broodstock (SS) at 11.8 and 18.4 DHW. Coral performance based on various heat tolerance traits has further been shown to both strongly and weakly correlate between rapid and moderate heat stress assays (*23*, *59*). These findings caution against the use of rapid stress assays to select stock for restoration. Furthermore, our results suggest Symbiodiniaceae traits, such as *F*_v_/*F*_m_, may not be appropriate when the aim is to detect variation in heat tolerance that has a host basis. Future research should focus on identifying phenotypic coral traits that are consistently indicative of heritable tolerance across populations. Particularly relevant would be to examine whether coral survival represents such a phenotype across coral species and geographic localities, as suggested by the work on *A. digitifera* in Palau (*23*). Alternatively, genetic markers of heat tolerance could be used for the purpose of selective breeding. However, the polygenic nature of coral heat tolerance has to date impeded the development such a genetic tool and may necessitate the usage of polygenic scores rather than individual biomarkers.

Genetically distinct populations can harbor different mechanisms to deal with high temperatures (*60*). Heat tolerance is highly polygenic in corals, and there are likely multiple pathways that enhance coral heat tolerance (*29*). Therefore, selective breeding of different broodstock based on heat tolerance phenotypes are expected to have different genetic outcomes and produce offspring that perform differently across traits under high temperatures, as was seen in this and previous studies. While population genetic data with adequate resolution to assess differentiation and genetic diversity between and within the populations at Davies and Moore Reef are currently not available for *A. spathulata*, population differentiation is unlikely to explain the differential enhancement of Davies and Moore Reef recruits seen here, since high levels of gene flow and minimal genetic differentiation have been observed across the region encompassing Moore and Davies reefs in other *Acropora* species (*30*, *34*). Alternatively, because sample sizes of the parental broodstock used for the crosses were small (*n* = 3 to 9), genetic differentiation may have occurred at the level of subpopulation (sampled broodstock), resulting in differences in selective breeding outcomes. The limited number of crosses within the RR, MM, and SS offspring groups—where three to four broodstock colonies were crossed—could have resulted in genotype effects that may be interpreted as cross effects. It is therefore recommended to maximize the number of colonies contributing to bulk fertilization reactions to minimize confounding genotypic effects, with the potential added benefit of enhancing genetic diversity and thus adaptive capacity of the stock.

Environmental variation can affect the heat tolerance of coral (*33*, *46*, *61*). The rapid heat stress assays of *A. spathulata* along the GBR identified that variation in historical high temperatures, thermal stress events, thermal fluctuations, and ocean currents correlated with heat tolerance variation (*46*). Correlation between heat tolerance phenotypes and environments can represent adaptation and/or acclimation in populations to their environments (*29*). The acclimation of broodstock to their environment poses a barrier to selective breeding based on phenotypes because it can mask the heritable genetic variation contributing to traits. Selective breeding based on phenotypes may result in limited enhancement of stock (e.g., survival and growth of the Moore Reef recruits were not enhanced here relative to all control groups through selective breeding) if nonheritable components are inadvertently targeted. It is worth noting that Davies [mean DHW (2014–2022) = 0.04, max DHW (2014–2022) = 1.62, MMM of 28.4°C] and Moore Reefs (mean DHW (2014–2022) = 0.20, max DHW (2014–2022) = 4.88, MMM of 28.7°C) have experienced different thermal environments (*34*). Different levels of heat exposure, light exposure, and other environmental variation could have resulted in site-specific acclimation and/or adaptation, potentially explaining the different outcomes of selective breeding between corals from Davies and Moore reefs (*34*, *46*).

The heat tolerance of selectively bred coral can also differ across life stages [i.e., larvae, recruit, adult; (*31*)]. This is perhaps expected, given that the gene expression of corals under temperature stress differs between life stages, suggesting the mechanisms conferring heat tolerance to a coral change over its life span (*62*). Nevertheless, selective breeding based on adult phenotypes has successfully enhanced recruit heat tolerance in some cases [here and in (*31*)], and therefore, genetic variants that affect heat tolerance similarly between life stages likely exist. These observations reflect the possibility for genes to have decoupled, synergistic, or antagonistic effects on heat tolerance between life stages (*63*). Furthermore, strong maternal effects can affect the performance of early coral life stages (*64*). From a practical perspective, these genetic and maternal effects—which we know little about—will affect the success of breeding for heat tolerance in corals, limit the ability to extrapolate the findings of this study to other life stages of *A. spathulata*, and highlight the need to test the performance of selectively bred corals through to their adult life stage and beyond a first generation.

### Heat-evolved symbionts enhanced bleaching resilience with trade-offs under ambient conditions

Inoculation with HE symbionts is a promising strategy for coral enhancement. Corals harboring HE symbionts have shown evidence of enhanced bleaching resilience (color and/or *F*_v_/*F*_m_): *A. spathulata* recruits (here), *Acropora kenti* spreviously referred to as *Acropora tenuis*; (*65*)] larvae (*42*) and recruits (*44*), adult *Galaxea fascicularis* (*43*), and the sea anemone *Exaiptasia diaphana* (*66*). However, not all HE strains confer enhanced bleaching resilience to coral (*36*, *42*). It has been shown that HE symbionts can increase thermal tolerance without compromising growth in adult corals (*43*) and recruits (*44*). However, here, inoculation with HE symbionts imposed a trade-off where recruits exhibited reduced growth under ambient conditions. Note that the experimental designs (e.g., length and extent of temperature stress) used to test HE symbionts have differed, and this may explain some of the variation in the results among studies, emphasizing the need to optimize and standardize protocols for testing assisted evolution strategies. However, assuming that mean gray values are proportional to symbiont densities and that WT and HE symbionts translocate carbon at equivalent rates, WT-treated recruits achieved a greater uptake of the inoculum and hence may have had more carbon available per unit host area for growth. Future studies should investigate if this trade-off can be accounted for through host-symbiont cell ratios and if the observation persists into later growth phases. Alternatively, the effect of HE symbionts may be dependent on host species or genotype. While *C. proliferum* has been identified to be a generalist species capable of living in a range of coral host species (*67*), host species or genotype may influence its performance in hospite and stock-dependent outcomes of inoculation with HE symbionts warrant further investigation.

### Combining interventions

We demonstrate that combining interventions can enhance recruit heat tolerance across traits, as it achieved greater enhancement in the survival of Davies Reef recruits under elevated temperatures than inoculation with HE symbionts alone (Fig. 9). However, the effect of interventions on the enhancement of coral stock when applied in combination cannot be assumed to be additive. For instance, there was little added benefit to selectively breeding the Moore Reef broodstock in addition to inoculating bulk-produced recruits with HE symbionts (Fig. 10). Combining interventions enhanced the survival of Moore Reef recruits under an elevated temperature in fewer instances (time points) than inoculation with HE symbionts did when used in isolation. Applying two interventions to the effect of one represents an inefficient use of resources. From another perspective, applying two interventions can act as assurance for achieving stock enhancement where the outcome of one (or multiple) interventions is uncertain. The complexity of the outcomes of combining interventions observed here highlights the need for further testing of interventions with common goals in combination.

### Management recommendations regarding the production of enhanced stock

We have found that selectively breeding corals based on broodstock *F*_v_/*F*_m_ responses under a short heat stress assay of 8 hours, and inoculating offspring with HE can enhance stock for restoration. However, this work simultaneously cautions against generalizations pertaining to interventions that have genotypic, population, trait, and environment specific outcomes. Further, measuring algal symbiont trait values (*F*_v_/*F*_m_)—which may or may not be affected by coral host genetic factors—to assess heritable variability in coral broodstock tolerance to summer heat waves may not be appropriate. Coral survival in response to longer-term heat stress, which is likely underpinned by many host and symbiont traits, may represent a more reliable trait to rank the thermal tolerance of broodstock earmarked for selective breeding (*23*), and this should be tested for additional coral species and locations. However, we acknowledge that long-term heat tolerance assays to select parental brood stock may not be scalable for restoration programs due to time and resource constraints. This highlights a key challenge in the development of assisted evolution interventions: making them effective at large scales. More broadly, the efficacy of assisted evolution interventions will depend on the substantial advancements that are being made in coral aquaculture and deployment technology (*68*, *69*) and the population genetic characteristics of the recipient population (e.g., effective population size).

The extrapolation of the results from this study must be conducted with care since they were obtained in a laboratory and used a single HE and WT strain; findings may differ when applied across reef environments. Given the variable outcomes of assisted evolution interventions, it is vital for management programs considering the production of enhanced stock to be specific as to their goals for stock performance. For example, a program should determine which coral life stage it is seeking to enhance since an intervention can have different outcomes in different life stages [selective breeding enhanced the performance of recruits but not larvae in (*31*)]. Given adult corals are more sensitive to high temperatures compared to juvenile corals (*50*, *51*) and are responsible for natural seeding of reefs, we recommend that enhancing adult heat tolerance be a priority. It is also necessary to consider which fitness traits are most important because interventions can induce trade-offs and different interventions can enhance different traits. Nonetheless, survival is paramount to fitness and has been successfully enhanced using assisted evolution interventions [for example, here and in (*23*, *43*)].

Furthermore, it would be remiss to not consider maximizing stock (host and Symbiodiniaceae) genetic diversity and then allowing natural selection on reefs to facilitate adaptation as an alternative restoration approach (*70*). It is likely that maximizing diversity is an optimal approach in degraded systems with small population sizes (*71*), and conservation aquaculture should equally ensure genetic diversity is maximized even in reef systems that are not (yet) genetically depauperate. Generally, conservation approaches must be tailored to the characteristics and needs of populations, species, and ecosystems. Therefore, selective breeding and inoculation with HE symbionts should continue to be investigated as tools for coral reef conservation/restoration and integrated with broader conservation efforts, including the reduction of global emissions.

## MATERIALS AND METHODS

### Coral collection and broodstock testing

The methods used for rapid heat stress assays of the broodstock are outlined in detail in (*34*) and (*46*) and briefly described here*. A. spathulata* was chosen as the study species due to its ecological role as a fast growing, reef-building coral (*72*), pan-tropical abundance (*73*), and amenability to being spawned in captivity. Two populations (from different reefs) were studied here to investigate variability in intervention outcomes between source reefs. Gravid, adult colonies were collected from the reef crest and upper reef slope prior to the December full moon in 2022; 18 *A. spathulata* were collected from Moore Reef (16.88802°S 146.18669°E and 16.88965°S 146.18745°E), and 20 *A. spathulata* colonies were collected from Davies Reef (18.82563°S 147.62608°E) on the GBR (Great Barrier Reef Marine Park Authority permit G22-47337.1). The Moore Reef and Davies Reef colonies were tested in different replicate runs of a rapid heat stress assay using the SeaSim-in-a-box (SSIAB) experimental system (*33*) on the Cape Ferguson research vessel. The rapid heat stress assays involved assigning one fragment of each of the colonies to each of 12 tanks at the MMM of the reef; the reef MMM values were calculated to be 28.4°C for Davies Reef and 28.7°C for Moore Reef using NOAA Coral Reef Watch Operational Daily Near-Real-Time Global 5-km Satellite sea surface temperature data. During the thermal stress assay, three replicate tanks were run at each of four temperature treatments: the reef MMM and +3°C, +6°C, and +9°C above the MMM. The tanks were ramped from the MMM to the target temperatures over a period of 3 hours, held at the target temperature for 3 hours, then ramped down to the MMM over 2 hours. Experimental runs began at 1100 AEST and light levels were maintained at 300 PAR (mmol photons/m^2^s^2^) and gradually turned on/off at sunrise/sunset. At the end of temperature ramp down to MMM, imaging pulse amplitude modulated (IPAM; Imaging PAM Maxi version, Walz, Germany) fluorometry was used to measure the photosynthetic performance of the coral using *F*_v_/*F*_m_ of the photosymbionts within the fragments. The IPAM was used with the following settings: Measuring light intensity = 2, frequency = 1, gain = 1, damping = 1, and saturation pulse = 7. *F*_v_/*F*_m_ data were used to calculate ED_50_ estimates for the colonies. The colonies were assigned performance rankings from 1 to 18 or 20 (depending on broodstock size) from most to least heat tolerant based on their ED_50_ values. The homoscedasticity of the *F*_v_/*F*_m_ ED_50_ values between Moore Reef and Davies Reef corals was tested using Levene’s tests and the “car” package (*74*). An analysis of variance (ANOVA) was then used to compare *F*_v_/*F*_m_ ED_50_ values between the broodstock reefs.

### Coral spawning and selective breeding

Broodstock colonies were held in outdoor aquarium systems in the National Sea Simulator (SeaSim) at the Australian Institute of Marine Science (AIMS) in 1120- and 290-liter aquarium systems under natural light. Individual colonies were isolated within the holding tanks using polyvinyl chloride (PVC) isolation chambers upon signs of spawning following the December full moon (07 December 2022). The isolation chambers allowed the spawn of individual colonies to be contained for collection so that controlled crosses could be conducted, with the added benefit of reducing handling that can induce stress to the broodstock. Crosses were limited to the colonies that spawned in synchrony on a given night. The egg-sperm bundles of the colonies were combined in bulk crosses according to broodstock heat tolerance (based on ED_50_ rankings) to produce four offspring groups with three to nine parental colonies (table S2) for each reef: (i) offspring of the most heat-tolerant broodstock with rankings in the top 25 percentile (RR), (ii) offspring of the moderately heat-tolerant broodstock with rankings between the 37.5 and 62.5 percentiles (MM), (iii) offspring of the most thermally sensitive broodstock with rankings in the bottom 25 percentile (SS), and (iv) offspring produced from a bulk cross containing gametes from all colonies that spawned on the night when the most colonies spawned in synchrony (Bulk). These crosses were compared to assess whether *F*_v_/*F*_m_ ED_50_ phenotyping under rapid heat stress assays could be used to enhance recruit tolerance under ecologically meaningful levels of heat stress. It was hypothesized that RR would outperform the three control offspring groups (SS, MM, and Bulk) under elevated temperature conditions. Each offspring group was produced by combining approximately equal volumes of egg-sperm bundles from the parent colonies in an 85-liter fertilization tank, which were agitated with air flow until bundles were broken apart. A volume of 5 to 10 ml of bundles from each colony were combined in a cross, equalized to the smallest volume of bundles produced from a colony. The gametes were then left to fertilize for ~30 min before the water flow to the tanks was turned on to remove excess sperm from the systems. The cultures of embryos were held in replicate 12- and 85-liter cones, initially maintained with low air and water flow, which was slowly increased once embryos surpassed the prawn chip developmental stage. Incoming seawater was filtered to 0.04 μm before being held in a storage reservoir within the SeaSim and then filtered to an additional 1 μm before entering the experimental system.

### Recruit settlement

Larvae were manually counted and pipetted (three to five larvae per well) between 7 and 15 days postfertilization into uniquely numbered PVC slides, each containing four 50-μl wells. Groups of four slides loaded with larvae from the same offspring group were placed in separate containers and given a settlement cue. Treating wells with ethanol-based *Titanoderma* sp. extract (*75*) and the addition of Hym-248 peptide [1 × 10^−6^ M; (*76*)] did not successfully induce settlement in the larvae. Desired settlement was achieved through adding a small volume of solution of crushed live CCA (unverified species taken from live rock in the SeaSim) in filter-sterilized seawater (FSW) to each well. Containers of slides were undisturbed for a minimum of 12 hours until successful settlement was observed. This was repeated to produce 32 to 78 replicate slides of each offspring group containing settled recruits for Davies Reef (herein referred to as Davies Reef recruits) and 37 to 45 replicate slides for Moore Reef (herein referred to as Moore Reef recruits); fewer slides of recruits were produced for offspring groups with limited numbers of larvae (table S2).

When most of the larvae had attached to the slides and begun metamorphosis, the containers were gently filled with FSW. The containers were left in a temperature-controlled room (28.4°C) for at least 24 hours to allow the offspring to fully attach (at which point they became settled coral recruits) before moving them. Note that some larvae cosettled as clumps and others as single individuals, which herein are referred to as clump and single recruits, respectively (fig. S4). This cosettlement behavior has previously been shown to influence the performance of *M. capitata* recruits and thus was incorporated into our analyses (*77*, *78*). Furthermore, larvae varied in their settlement position, such that they settled in the center (inside the well, not touching the well edge), on the edge (touching the well edge), or outside (outside the well, not touching the well edge) of the slide wells; settlement position was therefore also included in our analyses (fig. S4). Slides of recruits were placed into holders, referred to as “cassettes” (fig. S4). Between 10 and 13 cassettes were allocated to each of 16, 49-liter acrylic tanks at 28°C (fig. S4). A random number generator was used to randomly position the slides in tanks while ensuring an equal representation of each offspring group across all replicate tanks. The slides were maintained in the same tank position and vertical orientation throughout the experiment.

### Symbiont inoculations

To investigate whether recruit heat tolerance was enhanced via the inoculation of the recruits with HE Symbiodiniaceae of *C. proliferum*, eight of the tanks were inoculated with a WT strain (WT10, SCF055-01.10) and eight with a HE strain (SS8, SCF055-01.08) once a week for 5 weeks before the experiment (five inoculations, each conducted at a density of ~12,000 cells/ml). It was hypothesized that recruits inoculated with HE symbionts would perform better under elevated temperature conditions than those inoculated with WT symbionts since the HE symbiont strain used here has previously been used to enhance bleaching resilience in hospite (*43*, *44*, *66*). The tanks were drained to approximately half full (~20 liter) before the introduction of the inoculum, and water in-flow was turned off for at least 16 hours postinoculation to maximize the uptake of Symbiodiniaceae by the recruits. A no-inoculation control was not included in this experiment; however, phenotypic and molecular evidence supports successful uptake of the intended symbiont strains (see Results and Discussion).

### Recruit stress testing

A stress test was conducted to assess the effect of selective breeding and inoculation with HE symbionts on the heat tolerance of coral recruits under ecologically relevant levels of thermal stress. SeaSim provided a supervisory control and data acquisition (SCADA) system to automatically maintain the target water temperature in the experimental system by mixing streams of 22° and 36°C seawater. Temperature manipulation within the system was based on feedback measurements of the water as it entered the experimental room, recorded constantly, and averaged at 1-s intervals by the SCADA system. Water flow into the acrylic tanks was maintained at ~0.8 liter/min through plastic water lines. Incoming seawater from the ocean was filtered to the same specification as larval rearing tanks (0.04 μm before dam storage and then 1 μm immediately prior to entering the experimental system) to prevent Symbiodiniaceae from entering the tanks and contaminating the treatments. Hydra FiftyTwo HD light-emitting diode lights (Aquaria Illumination) were arranged above tanks to give uniform light with an average and environmentally realistic (*54*) intensity across the top of the cassettes of 100 μmol photons m^2^ s^−1^ that was measured using a LI-COR LI-250A PAR Meter with LI-192 sensor. Sunrise was adjusted to be later in the day, so that recruits remained dark adapted for fluorometry measurements, with lights set to turn on at 1100 and sunset beginning at 1800 with a 3-hour ramp time, i.e., lights were completely off by 2100. Cleaning was conducted weekly to reduce algal biomass within the tanks. Recruits were fed daily during the acclimation period and throughout the experiment, with each of the 16 tanks receiving 20 ml of algae mix (2000 cells/ml).

After 5 weeks of inoculation and acclimation to the experimental tanks, when the recruits were 51 to 59 days old (postfertilization) and pigmented (i.e., harbored high densities of symbionts), eight of the tanks were maintained at an ambient temperature (28°C), and eight were ramped to an elevated temperature (32°C) at a rate of 0.5°C/hour (*64*). Each combination of temperature (elevated or ambient) and Symbiodiniaceae (inoculated with HE or WT symbionts) treatments was represented across four replicate tanks. Variation in climate control within the room resulted in differences in the average flow-through tank water temperature of 31.6° to 32.5°C in the elevated tanks and 27.9° to 28.0°C in the ambient tanks as detected by manually recording in tank temperatures twice daily for a period of 14 days with a NIST Traceable calibrated thermometer; this variation was accounted for in analyses of the data collected in this experiment by including tank as a random effect in statistical models (see below). Degree heating week of 4°C may result in a risk of coral bleaching, and the DHW of 8°C is often associated with reef-wide coral bleaching (*47*). After 61 days of experimentation, the recruits from Davies (31 DHW) and Moore (29 DHW) Reef accumulated ~4-fold the heat stress known to elicit reef-wide coral bleaching and ~1.5-fold the level of heat stress that decimated the reefs of the Florida Keys in 2024 [22 DHW; (*79*)]; note that observations of coral bleaching are largely based on adult colonies that dominate coral cover. The recruits were therefore exposed to levels of heat stress recorded during marine heat waves that are known to elicit stress responses in corals, followed by levels projected for the GBR under certain SSPs (*49*).

### Recruit sampling and ITS2 analyses

ITS2 metabarcoding was used to discern whether the recruits acquired nontarget symbionts through contamination of the experiment with the CCA settlement cue or otherwise. Offspring groups with large sample sizes—RR and SS from Davies Reef—were sampled at 28 days, while the recruits in the ambient and elevated treatments were still pigmented, indicating that they contained Symbiodiniaceae. A single Davies Reef RR and SS recruit were sampled from each tank (*n* = 16 recruits per offspring group). Sampled recruits were snap frozen in liquid nitrogen and stored at −70°C.

DNA extractions were conducted on individual recruits in an isolated reaction using a modified salting-out method adapted from Wilson *et al.* (*80*), with DNA-blank controls included in the protocol to detect potential contamination during extractions (text S2). PCR amplification of the ITS2 region of the Symbiodiniaceae DNA was conducted in triplicate using the primers and protocol developed by Hume *et al.* (*81*). PCR products were pooled and indexing PCRs conducted (*82*). Indexing PCR products were purified using Beckman Coulter AMPure XP Bead-Based Reagent and quantified using a NanoDrop One Microvolume UV-Vis spectrophotometer. Samples were sequenced using an Illumina MiSeq v3 system at the Walter and Eliza Hall Institute (Melbourne, Australia). The sequence data were submitted to the SymPortal platform to identify ITS2-type profile representative of putative Symbiodiniaceae taxa (*83*).

The output from SymPortal was visually compared to a reference profile of SCF055-01.10 published in (*84*) to confirm that recruits had taken up the symbionts provided and were free from contamination. Sequences were trimmed at the 5′ (*n* = 3 nucleotides) and 3′ (*n* = 1 nucleotide) ends following the discovery of likely sequencing errors (single-nucleotide polymorphisms; see text S1).

### Imaging

Given assisted evolution interventions aim to enhance the fitness of organisms and various traits are expected to influence fitness to differing extents, we quantified multiple traits to assess the performance of the experimental groups. Images were taken to obtain data on recruit color [mean gray value, where a lighter color can indicate lower symbiont densities indicative of coral bleaching; (*85*)], growth, and survival before the temperature ramp and then after 25, 39, and 53 days of heat stress testing. Survival data were also collected from images taken at 61 days of heat stress testing. High-resolution photos were taken of the cassettes of slides with a mounted Nikon DSLR camera and Camera Control Pro software using the following settings: exposure mode set to aperture priority, 1/60-s shutter speed, f/14 aperture, 70-cm camera height, and a strobe intensity of 2.0. The mean gray value of a standard slide was measured in ImageJ for three representative images per tank and per time point (first, middle, and last images taken for each tank); the analysis of this data confirmed the lighting remained consistent throughout the experiment (text S3). Image analysis was semi-automated using the open-source machine learning program ilastik (*86*) following methods outlined in (*87*). Slide images were cropped from the cassette images using FastStone Image Viewer 7.7 (FastStone Image Viewer, 2022). Images that were representative of the slides within the experiment (15 to 25 images per Symbiodiniaceae and temperature treatment) were imported into ilastik and used for machine learning training. Machine learning training involved creating a Pixel classification project in ilastik to classify each pixel in the images into different categories based on their features. All available pixel features—including color, intensity, texture, and edge—were used to construct a model with an optimized ability to differentiate pixels belonging to recruits from pixels of other image components (*87*). The images were manually annotated using the “paintbrush” tool to highlight pixels associated with the class of interest—live recruits—and other classes such as slide, algae, and dead recruits. The manual annotations were used to build a machine learning model capable of identifying live recruits in the images by implementing a random forest algorithm. The model was applied to the training images to predict the class of each pixel, and the model performance was visualized using the “live update” tool. Retraining was conducted until the model could be used to accurately (>95%) differentiate between recruits and other components of the images. The optimized model was applied to the remaining dataset, allowing for a consistent and automated discernment of recruits from the images. Batch processing of these images generated an output of simple semantic segmentation masks for each image which, together with the raw slide images and a customized script (*87*), was used to measure the survival, growth, and color of the recruits in Fiji ImageJ (*88*). The automatically identified recruits were manually reviewed and verified, and individual recruits were tracked through time. In instances where the model’s selections were incorrect, recruit phenotypes were manually remeasured in ImageJ. This was necessary due to recruits not being identified or image components being incorrectly identified as recruits, in instances such as when recruits were in proximity with one another or other biological material was on the slides. To measure recruit growth and account for differences in starting size, the fold change in area of each recruit was calculated by dividing the area of the recruits at later time points by the area of the recruit before the temperature ramp; this metric (herein referred to as growth) therefore reflects proportional growth rather than absolute growth. To measure the color, the additive inverse of mean gray value was calculated for each recruit, such that higher values were indicative of darker (less bleached) recruits.

### Fluorometry

A Walz IPAM fluorometer was used to collect dark adapted *F*_v_/*F*_m_ measurements—an indicator of photosynthetic health in the Symbiodiniaceae, where higher values are indicative of better performance, between 8:00 and 11:00 (before sunrise) before the temperature ramp and then after 25, 40, and 54 days of heat stress testing. The IPAM was used with the following settings: Image correction = MAXI, measuring light intensity = 2, frequency = 1, gain = 1, damping = 2, and saturation pulse = 7. IPAM surfaces were rinsed with fresh water and wiped down with ethanol in between taking measurements from each tank to prevent cross-contamination between Symbiodiniaceae treatments. Walz ImagingWin software was used to extract the *F*_v_/*F*_m_ values for each recruit at each sampling time point. Live recruits partially or fully obscured by algae were excluded to avoid nontarget fluorescence signals being included in the dataset.

### Statistical analyses of recruit phenotypes

Statistical modeling was conducted using R version 4.2.2 (*89*) and RStudio. Graphics were produced using the R package ggplot2 (*90*).

Note that all phenotypic measurements were taken from surviving recruits. Therefore, our analyses do not distinguish between the capacity of recruits to avoid bleaching and their ability to recover from bleaching; here, we use the term bleaching resilience to collectively refer to both traits. Bayesian models were run on each performance metric and linkage among the traits was not assessed; growth was modeled against a Gamma distribution (log-link), survival against a Bernoulli (logit link), and *F*_v_/*F*_m_ and mean gray value data against a Student’s *t* (identity link). All models included the population effects of offspring group, Symbiodiniaceae treatment, sampling time point (discrete), temperature treatment (discrete), and broodstock reef (along with their interactions) as well as the varying effects of recruits nested within slides nested within tanks. Preliminary analyses (text S4) indicated that whether the recruits were clumps or singles (settlement behavior) affected recruit growth, *F*_v_/*F*_m_, and color, but not survivorship. The effect of recruit settlement behavior on growth and color was dependent on temperature treatment and time point and the effect of recruit settlement behavior on *F*_v_/*F*_m_ was exclusively dependent on temperature; hence, the nested and varying effects of these variables were included in the Bayesian models analyzing these traits (text S4). The inclusion of settlement behavior in growth models was further warranted because initial size differences among offspring groups exist due to variation in settlement behavior, with some groups containing a higher proportion of clumped recruits (text S4). Initial recruit sizes were additionally compared among Symbiodiniaceae treatments using an ANOVA to confirm equivalence before the temperature ramp. Further preliminary analyses (text S5) indicated that settlement position affected each performance metric in a manner that was dependent on temperature treatment and time point, such that the nested and varying effects of settlement position were also included in the Bayesian models. Weakly informative priors were applied to all parameters. All Bayesian models were fit using the brms package (*91*). The models were run using three parallel chains with a 1000 iteration warmup, 5000 iterations, and a thinning interval of 5. The models were found to be well mixed and to have converged upon a stable posterior (all $\hat{R}$values <1.01), all parameters had effective sample sizes exceeding 1000 and efficiencies exceeding 0.5. Posterior predictive checks and simulated residuals indicated that the models had adequate goodness of fit. While the models predicting growth did not achieve a strong fit, the Gamma models provided an adequate approximation to the observed data and were not outperformed by the Gaussian and lognormal alternatives (text S6).

Specific contrasts were defined to tease apart interactions and explore pairwise differences among main treatment effects. Note that within this design, Bulk crosses represent stock produced using methods mirroring typical batch coral production that would be made in the absence of assisted evolution of the coral host via a fertilization reaction of all broodstock colonies, regardless of heat tolerance. Furthermore, Bulk inoculated with WT symbionts (not heat-evolved) symbionts represent stock produced using cultured symbionts that have not been subjected to assisted evolution. The performance of RR inoculated with WT symbionts was therefore compared to that of the control offspring groups (Bulk, MM, and SS) inoculated with WT symbionts for each reef, under each temperature treatment, and at each time point to assess the effects of selective breeding. The performance of the Bulk offspring groups inoculated with WT symbionts and HE symbionts were similarly compared to assess the effects of inoculation with heat-evolved symbionts. To assess the effects of combining interventions, the performance of RR inoculated with HE symbionts was compared to that of Bulk with WT. Last, to compare the effect of applying the interventions in isolation and in tandem, we compared the relative performance of RR inoculated with WT symbionts (representing the product of selective breeding), Bulk inoculated with HE symbionts (representing the product of inoculation with HE), and RR inoculated with HE symbionts (representing the product of applying both interventions in tandem) against the baseline of the control group of Bulk inoculated with WT. Posteriors of each contrast were then summarized by the 95% highest probability density (HPD) intervals and central tendency (median or mean depending on the model). The HPD and the proportion of the posterior below and above zero (posterior probability) for the effect size of each pairwise comparison were graphed. The code used for data analysis (which contains complete model descriptions) is available at https://github.com/open-AIMS/Recruit-Assisted-Evolution, and data are stored on the AIMS data repository.

## Supplementary Materials

### This PDF file includes:

Supplementary Text S1 to S6 Figs. S1 to S5 Tables S1 to S4 References
