# Supplementary material for: Assisted evolution of corals and their symbionts enhances recruit heat tolerance but with complex outcomes
Source: Sci Adv. 2026 Jul 1;12(27):eaeb5575. doi: 10.1126/sciadv.aeb5575 (PMC13322255; doi:10.1126/sciadv.aeb5575)
Supplement: Supplementary file 1 — Supplementary Text S1 to S6 Figs. S1 to S5 Tables S1 to S4 References [file sciadv.aeb5575_sm.pdf]

Science Advances
AAAS

# Supplementary Materials for

## Assisted evolution of corals and their symbionts enhances recruit heat tolerance but with complex outcomes

Annika M. Lamb *et al.*

Corresponding author: Annika M. Lamb, a.lamb@aims.gov.au

*Sci. Adv.* **12**, eaeb5575 (2026)
DOI: 10.1126/sciadv.aeb5575

**This PDF file includes:**

Supplementary Text S1 to S6
Figs. S1 to S5
Tables S1 to S4
References

**Supplementary Text 1**

Unreliable sequence removal

Initial inspection of the ITS2 data showed the presence of some sequences that were not consistent with the reference profile. Further investigation revealed that they were identical to reference profile sequences except for a single nucleotide substitution or insertion at the first or second position. These unexpected sequences also occupied a proportion of the sample that seemed to be missing from the reference profile sequences we did have. For example, C1 is expected to make up 59-65% of a sample containing our inoculum. In one affected sample C1 occupied only 38%, and the most prevalent of these unexpected sequences, C1os occupied 25% of the sample (Fig. S1). The most likely explanation for these unexpected sequences is that they were caused by an error during the sequencing process. This is supported by the location of the SNPs within the first three nucleotides, and the multicopy nature of the ITS2 gene, meaning these mutations would have had to alternatively occur multiple times at the same location. To address these errors, the sequences were realigned in R using the AlignSeqs function from DECIPHER (*92*). The aligned sequences were then trimmed, with 3 nucleotides being removed from the 5’ end and 1 nucleotide removed from the 3’ end (Table S1, Fig. S2). After trimming all sequences were compared, and those that had become identical were assigned whichever sequence name was most prevalent in the dataset (Table S1). Application of this method was successful in removing the unexpected sequences and revising the reference profile sequences to their anticipated proportions (Fig. S1).

**Supplementary Text 2.**
DNA extraction protocol

This extraction method provides appropriate gDNA for microbial community 16S Mi-Seq/Next-Seq analysis. The protocol is adapted from Wilson *et al.* (*80*) and the reagents are listed in Tables S3 and S4.

Method:

1. Use forceps to transfer about 20 mg or 1-2 'match head size pieces' of tissue to paper towel. Rinse forceps in MilliQ water and then 80% EtOH between samples. Air dry tissue for 10 minutes.
   **If using frozen tissue, keep frozen until step 5.*

2. Prepare the primary extraction buffer (PEB) and divide equally into 2 x 50 mL falcon tubes (for this protocol, 2 x 37.5 mL). Add 507 µL of 10mg/mL lysozyme (~5 µL lysozyme per sample) to one of the Falcon tubes containing PEB and mix gently. This is now buffer PEBL.

3. Dispense ~20-30 acid washed glass beads (Sigma G1152 -100G, 710-1180uM) to the required number of pre-labelled 1.5mL microfuge tubes.

4. Aliquot 380 µL of the PEB/lysozyme mix (buffer PEBL) into the tubes containing glass beads.

5. Add air-dried or frozen tissue.

6. Homogenise in MD Biomedicals FastPrep-24 5G (or equivalent) for 2 x 40 seconds at 4ms.

7. Incubate homogenised samples at 37°C for 30 minutes.
   **For this experiment a programmable heat block was used.*

8. Combine the remaining 37.5 mL PEB with 2.0 mL of 20 mg/mL Proteinase K (~20µL Proteinase K per sample), this is now buffer PEBP.

9. Remove samples from the heat block and add 380 µL of the PEB/ Proteinase K (buffer PEBP) to each sample and mix well.

10. Incubate in a heat block at 65°C for 1-2 hours.
    **Ensure incubation times are consistent between batches of samples.*

11. Add 189.5 µL of 5M KOAc to each tube and mix well.

12. Incubate on ice for 30 minutes.

13. Spin at maximum speed for 15 minutes in bench top centrifuge at 8°C.

14. Transfer ~800 µL supernatant to fresh, pre-labelled 1.5mL microfuge tubes.

15. Add isopropanol (0.8 X vol= ~640 µL) to supernatant, to precipitate DNA. Mix gently.

16. Centrifuge at max speed for 12 minutes at 8°C.

17. Carefully remove and discard supernatant, leaving pellet.

18. Add 200 µL of 70% ethanol to wash the pellet and gently mix.

19. Centrifuge at max speed for 3 minutes at 8°C.
20. Remove and discard supernatant with extreme care.
    **use a low-volume pipette as not to disturb the pellet.*
21. Air-dry the pellet for 20-30 minutes.
22. Resuspend pellet in 35µl of Milli Q water and leave DNA at 4$^{o}$C overnight.
    **Resuspend in a solution volume appropriate for the DNA yield.*
23. Check samples by running 1 µL DNA with 3µL 1:3 diluted ‘Orange G’ dye on a 1.0% agarose / 1x TAE gel at 100V for ~30 minutes alongside uncut Lambda DNA as a size marker.

**Supplementary Text 3**.
Colour standardization

The mean grey value of the same section of the scale slide was measured in ImageJ for three images per tank, per timepoint (first, middle, and last images taken) to determine whether there were any differences in image lighting across the experiment due to potential changes in strobe intensity that would affect the reliability of recruit colour measurements. Changes in light were limited by charging the strobe batteries halfway through each photography session. The absolute difference in mean grey value of the slides was <15%, indicating that the lighting remained relatively consistent throughout experiment, making it unnecessary to scale each value to the Coral Watch Colour Chart (*93*), which was present in each image as a reference, in case scaling was required. One outlier grey value of the scale slide was detected using the rstatix package (*94*) in R and colour data from the photograph was omitted.

**Supplementary Text 4**.
Effect of settlement behaviour on recruit performance

The effect of whether the recruits co-settled in clumps (clumps) or as single individuals (singles) on the survival, growth, colour (additive inverse of mean grey value), and photochemical efficiency ($F_v/F_m$) of the recruits in ambient and elevated conditions across the timepoints of the 61 day-long experiment was assessed.

Methods
Bayesian modelling was conducted for each of the performance metrics using three parallel chains with a 1000 iteration warmup and 4000 iterations (*91*). The models were considered valid and interpreted if the bulk and tail estimated sample sizes were greater than 1000, Rhat values were 1.0, the posterior distributions of the estimates were normally distributed and unimodal, and the chains of the models tracked one another through time. The offspring groups varied in their settlement behaviour such that proportion of settled recruits in clumps ranged from 0.17 – 0.40. Selective breeding offspring group nested within broodstock population (Moore or Davies Reef) was therefore included as a random effect in the models, as were recruit unique identifier (to account for the repeated measures design) nested within slide nested within experimental tank. The emmeans package was used to conduct post hoc analyses of the estimated marginal means (EMM) estimated from the survivorship, colour, growth, and $F_v/F_m$ data to compare the performance of clumps and singles (*48*).

Results
Clumps had similar survival to singles under ambient and elevated conditions (95% highest posterior densities (HPDs) of odds ratios (OR) overlapped with one). Clumps had grown less than singles after 39 (OR = 0.86, HPD = 0.81 – 0.91) and 53 (OR = 0.85, HPD = 0.79 – 0.91) days in the elevated treatment but similarly to singles under ambient conditions and at 25 days under elevated conditions (HPDs of ORs overlapped with one). Clumps had higher $F_v/F_m$ than singles under ambient conditions after 25 (difference in estimated marginal means (ΔEMM) = 0.010, HPD = 0.005 – 0.015), 40 (ΔEMM = 0.013, HPD = 0.008 – 0.018), and 54 (ΔEMM = 0.011, HPD = 0.006 – 0.016) days but similar $F_v/F_m$ to singles under elevated conditions (HPDs of ΔEMMs overlapped with zero). Clumps were darker than singles after 25 (ΔEMM = 2.68, HPD = 0.41 – 5.19), 39 (ΔEMM = 3.48, HPD = 1.41 – 6.03), and 53 (ΔEMM = 3.50, HPD = 1.11 – 6.00) days under ambient but were similarly coloured to singles under elevated conditions (HPDs of ΔEMMs overlapped with zero).

**Supplementary Text 5**.
Effect of settlement position on recruit performance

The effect of whether the larvae settled in the centre (inside the well, not touching the well edge; referred to as centre recruits), on the edge (touching the well edge; referred to as edge recruits), or outside (outside the well, not touching the well edge; referred to as outside recruits), of the slide wells on the survival, growth, colour (additive inverse of mean grey value), and photochemical efficiency ($F_v/F_m$) of the recruits in ambient and elevated conditions across the timepoints of the 61 day-long experiment was assessed.

Methods

Bayesian modelling was conducted for each of the performance metrics using three parallel chains with a 1000 iteration warmup and 4000 iterations (*91*). The models were considered valid and interpreted if the bulk and tail estimated sample sizes were greater than 1000, Rhat values were 1.0, the posterior distributions of the estimates were normally distributed and unimodal, and the chains of the models tracked one another through time. The offspring groups varied in their settlement position such that proportion of centre, edge, and outside recruits ranged from 0.17 – 0.66, 0.26 – 0.68, and 0.03 – 0.15, respectively. Therefore, selective breeding offspring group nested within broodstock population (Moore or Davies Reef) was included as a random effect in the models, as were recruit unique identifier (to account for the repeated measures design) nested within slide nested within experimental tank. The emmeans package was used to conduct post hoc analyses of the estimated marginal means (EMM) estimated from the survivorship, colour, growth, and $F_v/F_m$ data to compare the performance recruits based on their settlement position (*48*).

Results

Centre (odds ratio (OR) = 2.32 x $10^6$, 95% highest posterior density (HPD) of OR = 90.94 – 2.03 x $10^9$) and edge recruits (OR = 24.28 x $10^4$, HPD of OR = 3.16 – 2.05 x $10^7$) had greater survival than outside recruits after 61 days under elevated conditions. Recruits that settled in different positions otherwise had similar survival (HPDs of ORs overlapped with one). Centre recruits had grown less than edge recruits after 53 days under ambient conditions (OR = 0.92, HPD = 0.87 – 0.97) but more than edge recruits after 53 days under elevated conditions (OR = 1.12, HPD = 1.04 – 1.19). Otherwise, centre, edge, and outside recruits had similar growth (HPDs of ORs overlapped with one). Centre recruits had higher $F_v/F_m$ than edge recruits after 25 (difference in estimated marginal means (ΔEMM) = 0.029, HPD = 0.024 – 0.033), 40 (ΔEMM = 0.029, HPD = 0.024 – 0.033), and 54 (ΔEMM = 0.025, HPD = 0.020 – 0.029) days under ambient conditions but similar $F_v/F_m$ to edge recruits under elevated conditions (HPDs of ΔEMMs overlapped with zero). Centre recruits had higher $F_v/F_m$ than outside recruits after 25 (ΔEMM = 0.052, HPD = 0.043 – 0.060), 40 (ΔEMM = 0.052, HPD = 0.043 – 0.061), and 54 (ΔEMM = 0.043, HPD = 0.034 – 0.052) days under ambient conditions, and 40 (ΔEMM = 0.052, HPD = 0.043 – 0.061) and 54 (ΔEMM = 0.043, HPD = 0.034 – 0.052) but not 25 (ΔEMM = 0.012, HPD = 0.000 – 0.055) days under elevated conditions. Edge recruits had higher $F_v/F_m$ than outside recruits after 25 (ΔEMM = 0.023, HPD = 0.014 – 0.031), 40 (ΔEMM = 0.024, HPD = 0.014 – 0.032), and 54 (ΔEMM = 0.019, HPD = 0.009 – 0.027) days under ambient conditions, and 40 (ΔEMM = 0.036, HPD = 0.024 – 0.049) and 54 (ΔEMM = 0.023, HPD = 0.004 – 0.039) but not 25 (ΔEMM = 0.006, HPD = -0.006 – 0.018) days under elevated conditions. Centre recruits were darker than edge recruits after 53 days under ambient conditions (ΔEMM 4.61, HPD = 2.29 – 6.92) and 25 (ΔEMM 6.90, HPD = 3.96 – 9.52) and 39 (ΔEMM 4.78, HPD = 2.07 – 7.58) days under elevated conditions but were otherwise similar in colour to edge recruits during the heat

treatments (HPDs of ΔEMMs overlapped with zero). Centre recruits (ΔEMM 3.79 – 22.74, lower HPD = 0.161 – 17.62, upper HPD = 7.43 – 28.92) and edge recruits (ΔEMM 8.30 – 17.96, lower HPD = 4.77 – 12.48, upper HPD = 11.96 – 23.27) were darker than outside recruits at all timepoints during the heat treatments.

**Supplementary Text 6.**
Modelling recruit growth

The growths of Davies and Moore Reef recruits were modelled separately using three alternative distributions: Gamma, Gaussian, and lognormal. For all six models, the dispersion of the residuals did not deviate significantly from expectations (DHARMa dispersion test, $P > 0.05$). However, the uniformity of residuals differed from the expected distribution in each case (Kolmogorov–Smirnov test, $P < 0.05$). While none of the models achieved a perfect fit, the Gamma models provided an adequate approximation to the observed data and was not outperformed by the Gaussian and lognormal alternatives (Fig. S5).

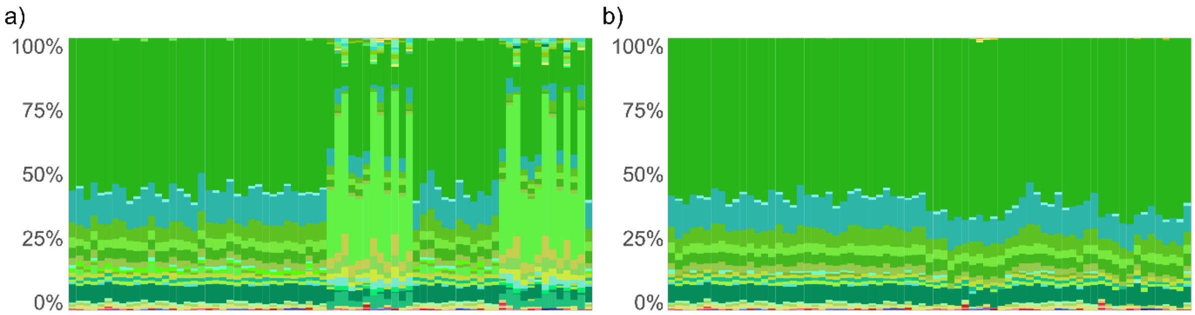


**Figure S1. Relative abundance of ITS2 DNA sequences from coral recruits**. Each bar represents an individual sample, and the colours they are comprised of represent the abundance of each sequence. a) Samples before trimming. b) Samples after trimming.

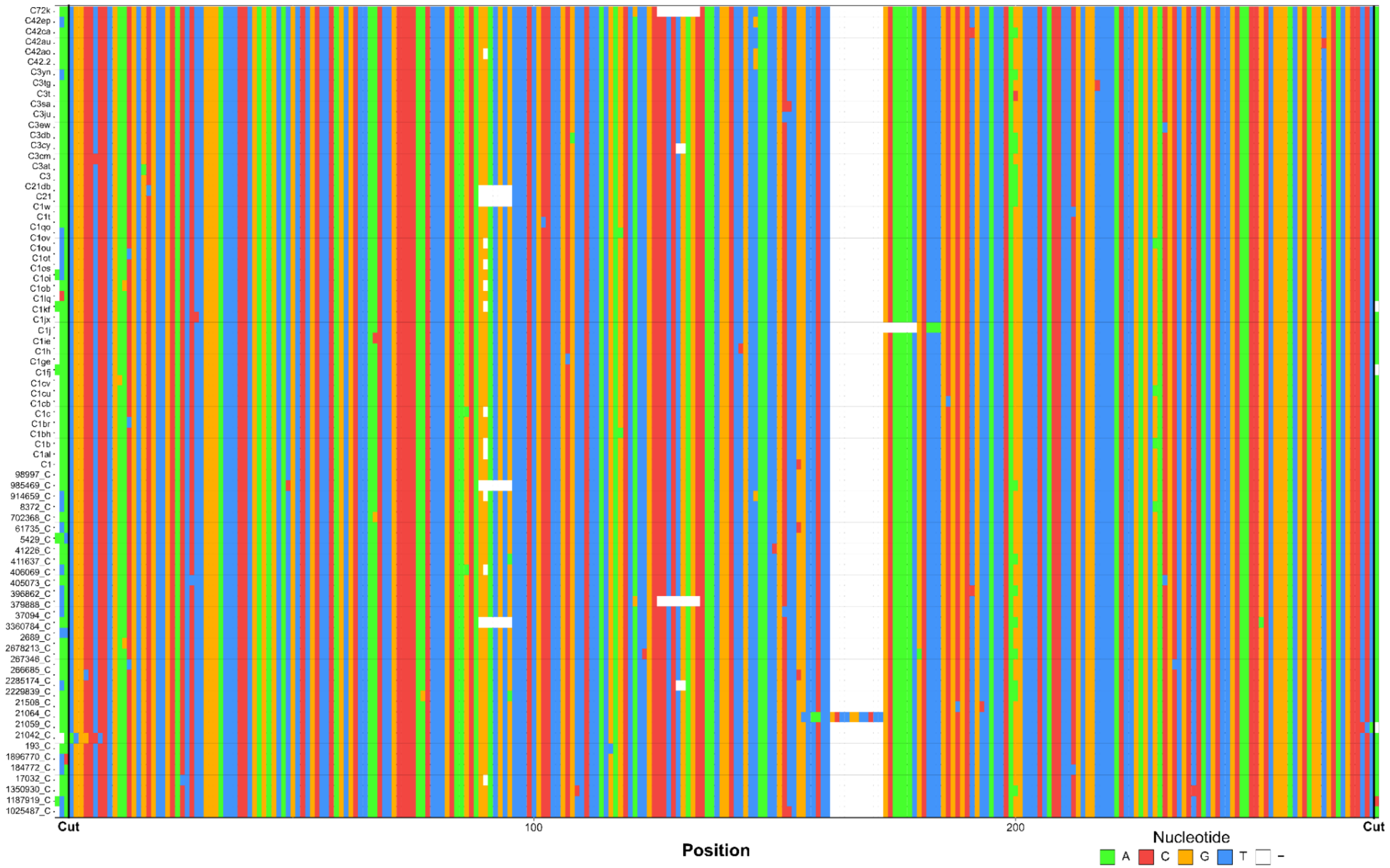


**Figure S2. Alignment of ITS2 sequences prior to trimming.** Trimming removed all nucleotides before the 4th position and all nucleotides after the 274th position.

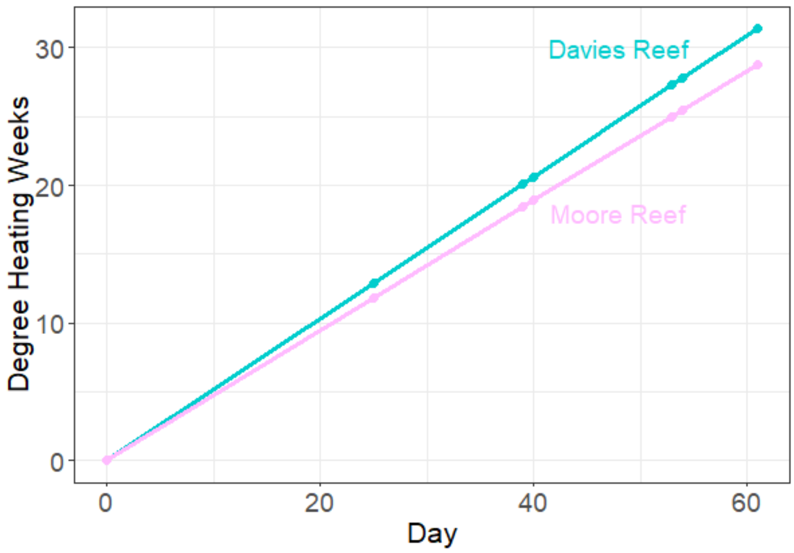


**Figure S3. Graph depicting the accumulation of thermal stress in degree heating weeks (DHW) for recruits in the elevated temperature treatments.** The offspring of broodstock from Davies Reef (maximum monthly mean (MMM) = 28.4°C) accumulated ~3.6 DHW per week (teal line) and the offspring of broodstock from Moore Reef (MMM = 28.7°C) accumulated ~3.3 DHW per week in the elevated treatment (pink line). Points along the line represent sampling timepoints.

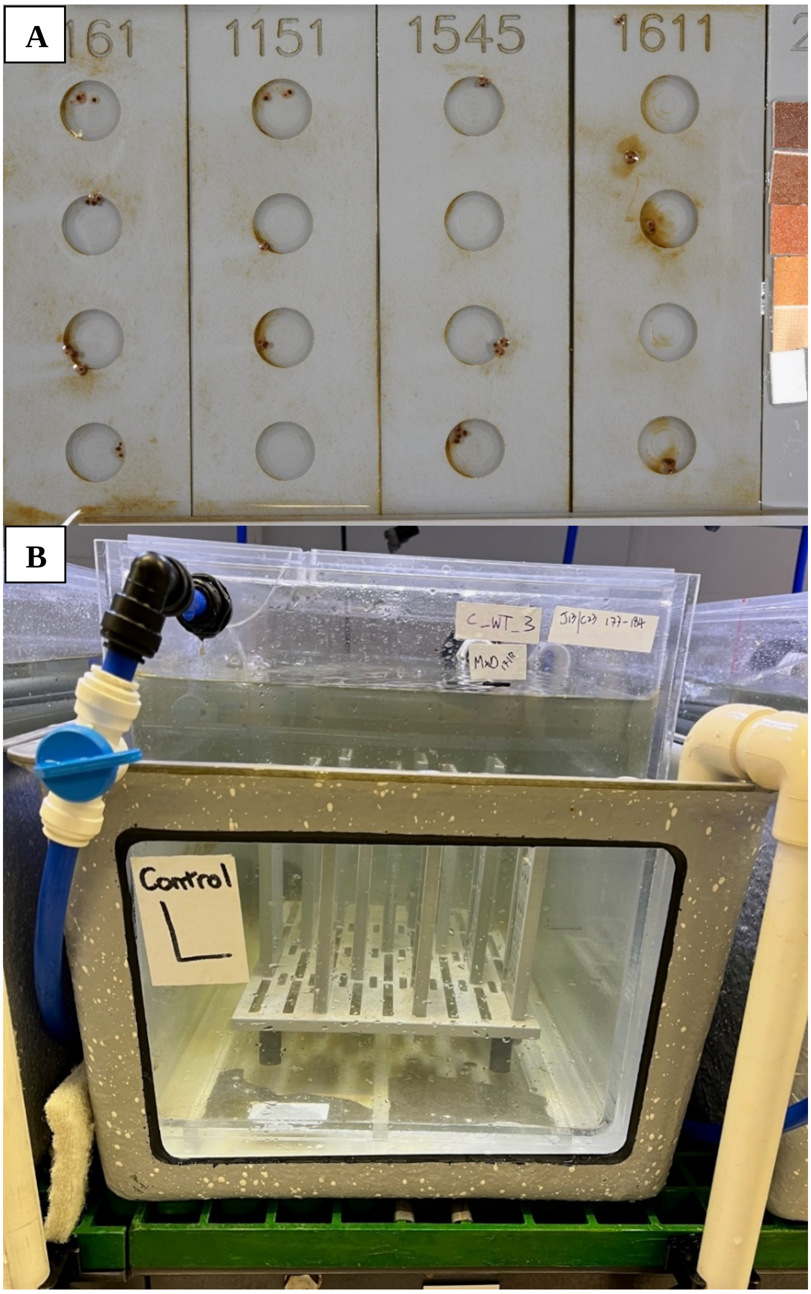


**Figure S4. Experimental setup.** A) Photograph of slides in cassettes with Coral Watch chart on right, used for physiological measurements (survival, colour, and growth). B) Cassettes within experimental tank system showing water jacket and vertical positioning of cassettes.

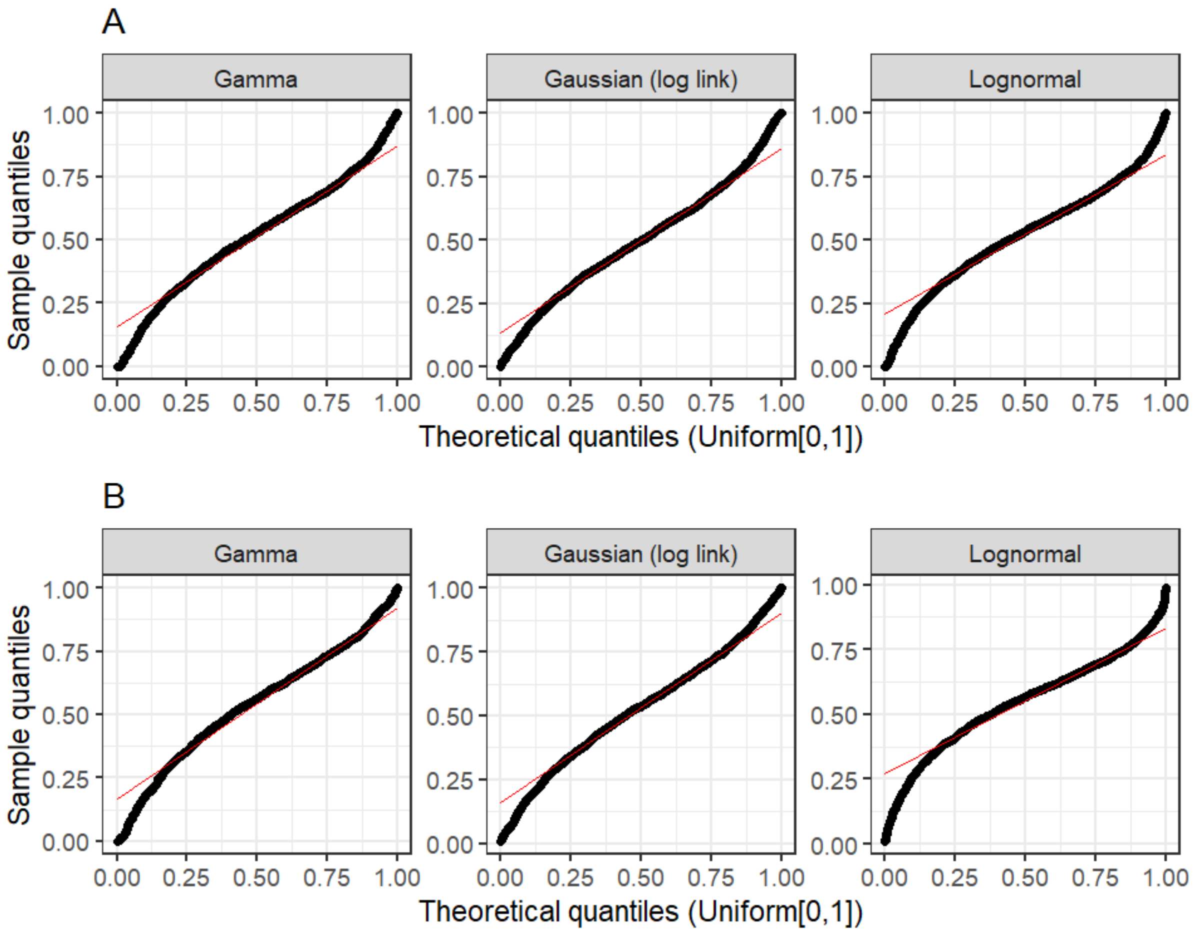


**Figure S5. Fit of Bayesian models estimating recruit growth.** Quantile–quantile (QQ) plots of scaled residuals from models of Davies (row A) and Moore Reef (row B) recruit growth fitted with Gamma, Gaussian (log link), and lognormal error distributions.

**Table S1. Trimmed sequences for ITS2 metabarcoding analyses**. Initial sequences and initial names are before trimming, and final sequence and final names are after trimming.

| Initial Name | Initial Sequence | Final Name | Final Sequence |
|---|---|---|---|
| **C1ge** | AAATGGCCTCCTGAACGTGCGTTGCACTCTTGGGATTTCCTGAGAGTATGTCTGC<br>TTCAGTGCTTAACTTGCCCCAACTTTGCAAGCAGGATGTGTTTCTGCCTTGCGTT<br>CTTATGAGCTATTGCCCTCTGAGCCAATGGCTTGTTAATTGCTTGGTTCTTGCAA<br>AATGCTTTGCGCGCTGTTATTCAGGTTTCTACCTTCGTGGTTTTACTTGAGTGAC<br>GCTGCTCATGCTTGCAACCGCTGGGATGCAGGTGCATGCCTCT | **C1** | TGGCCTCCTGAACGTGCGTTGCACTCTTGGGATTTCCTGAGAGTATGTCTGCTT<br>CAGTGCTTAACTTGCCCCAACTTTGCAAGCAGGATGTGTTTCTGCCTTGCGTTC<br>TTATGAGCTATTGCCCTCTGAGCCAATGGCTTGTTAATTGCTTGGTTCTTGCAAA<br>ATGCTTTGCGCGCTGTTATTCAGGTTTCTACCTTCGTGGTTTTACTTGAGTGACG<br>CTGCTCATGCTTGCAACCGCTGGGATGCAGGTGCATGCCTCT |
| **C1os** | ATATGGCCTCCTGAACGTGCGTTGCACTCTTGGGATTTCCTGAGAGTATGTCTGC<br>TTCAGTGCTTAACTTGCCCCAACTTTGCAAGCAGGATGTGTTTCTGCCTTGCGTT<br>CTTATGAGCTATTGCCCTCTGAGCCAATGGCTTGTTAATTGCTTGGTTCTTGCAA<br>AATGCTTTGCGCGCTGTTATTCAGGTTTCTACCTTCGTGGTTTTACTTGAGTGAC<br>GCTGCTCATGCTTGCAACCGCTGGGATGCAGGTGCATGCCTCTA | **C1** | TGGCCTCCTGAACGTGCGTTGCACTCTTGGGATTTCCTGAGAGTATGTCTGCTT<br>CAGTGCTTAACTTGCCCCAACTTTGCAAGCAGGATGTGTTTCTGCCTTGCGTTC<br>TTATGAGCTATTGCCCTCTGAGCCAATGGCTTGTTAATTGCTTGGTTCTTGCAAA<br>ATGCTTTGCGCGCTGTTATTCAGGTTTCTACCTTCGTGGTTTTACTTGAGTGACG<br>CTGCTCATGCTTGCAACCGCTGGGATGCAGGTGCATGCCTCT |
| **C1ob** | CATGGCCTCCTGAACGTGCGTTGCACTCTTGGGATTTCCTGAGAGTATGTCTGCT<br>TCAGTGCTTAACTTGCCCCAACTTTGCAAGCAGGATGTGTTTCTGCCTTGCGTTC<br>TTATGAGCTATTGCCCTCTGAGCCAATGGCTTGTTAATTGCTTGGTTCTTGCAAA<br>ATGCTTTGCGCGCTGTTATTCAGGTTTCTACCTTCGTGGTTTTACTTGAGTGACG<br>CTGCTCATGCTTGCAACCGCTGGGATGCAGGTGCATGCCTCTA | **C1** | TGGCCTCCTGAACGTGCGTTGCACTCTTGGGATTTCCTGAGAGTATGTCTGCTT<br>CAGTGCTTAACTTGCCCCAACTTTGCAAGCAGGATGTGTTTCTGCCTTGCGTTC<br>TTATGAGCTATTGCCCTCTGAGCCAATGGCTTGTTAATTGCTTGGTTCTTGCAAA<br>ATGCTTTGCGCGCTGTTATTCAGGTTTCTACCTTCGTGGTTTTACTTGAGTGACG<br>CTGCTCATGCTTGCAACCGCTGGGATGCAGGTGCATGCCTCT |
| **61735_C** | AATTGGCCTCCTGAACGTGCGTTGCACTCTTGGGATTTCCTGAGAGTATGTCTGC<br>TTCAGTGCTTAACTTGCCCCAACTTTGCAAGCAGGATGTGTTTCTGCCTTGCGTT<br>CTTATGAGCTATTGCCCTCTGAGCCAATGGCTTGTTAATTGCTTGGTTCTTGCAA<br>AATGCTTTGCGCGCTGTTATTCAGGTTTCTACCTTCGTGGTTTTACTTGAGTGAC<br>GCTGCTCATGCTTGCAACCGCTGGGATGCAGGTGCATGCCTCTA | **C1** | TGGCCTCCTGAACGTGCGTTGCACTCTTGGGATTTCCTGAGAGTATGTCTGCTT<br>CAGTGCTTAACTTGCCCCAACTTTGCAAGCAGGATGTGTTTCTGCCTTGCGTTC<br>TTATGAGCTATTGCCCTCTGAGCCAATGGCTTGTTAATTGCTTGGTTCTTGCAAA<br>ATGCTTTGCGCGCTGTTATTCAGGTTTCTACCTTCGTGGTTTTACTTGAGTGACG<br>CTGCTCATGCTTGCAACCGCTGGGATGCAGGTGCATGCCTCT |
| **1187919_C** | ATATGGCCTCCTGAACGTGCGTTGCACTCTTGGGATTTCCTGAGAGTATGTCTGC<br>TTCAGTGCTTAACTTGCCCCAACTTTGCAAGCAGGATGTGTTTCTGCCTTGCGTT<br>CTTATGAGCTATTGCCCTCTGAGCCAATGGCTTGTTAATTGCTTGGTTCTTGCAA<br>AATGCTTTGCGCGCTGTTATTCAGGTTTCTACCTTCGTGGTTTTACTTGAGTGAC<br>GCTGCTCATGCTTGCAACCGCTGGGATGCAGGTGCATGCCTCTC | **C1** | TGGCCTCCTGAACGTGCGTTGCACTCTTGGGATTTCCTGAGAGTATGTCTGCTT<br>CAGTGCTTAACTTGCCCCAACTTTGCAAGCAGGATGTGTTTCTGCCTTGCGTTC<br>TTATGAGCTATTGCCCTCTGAGCCAATGGCTTGTTAATTGCTTGGTTCTTGCAAA<br>ATGCTTTGCGCGCTGTTATTCAGGTTTCTACCTTCGTGGTTTTACTTGAGTGACG<br>CTGCTCATGCTTGCAACCGCTGGGATGCAGGTGCATGCCTCT |
| **1896770_C** | TCTGGCCTCCTGAACGTGCGTTGCACTCTTGGGATTTCCTGAGAGTATGTCTGCT<br>TCAGTGCTTAACTTGCCCCAACTTTGCAAGCAGGATGTGTTTCTGCCTTGCGTTC<br>TTATGAGCTATTGCCCTCTGAGCCAATGGCTTGTTAATTGCTTGGTTCTTGCAAA<br>ATGCTTTGCGCGCTGTTATTCAGGTTTCTACCTTCGTGGTTTTACTTGAGTGACG<br>CTGCTCATGCTTGCAACCGCTGGGATGCAGGTGCATGCCTCTA | **C1** | TGGCCTCCTGAACGTGCGTTGCACTCTTGGGATTTCCTGAGAGTATGTCTGCTT<br>CAGTGCTTAACTTGCCCCAACTTTGCAAGCAGGATGTGTTTCTGCCTTGCGTTC<br>TTATGAGCTATTGCCCTCTGAGCCAATGGCTTGTTAATTGCTTGGTTCTTGCAAA<br>ATGCTTTGCGCGCTGTTATTCAGGTTTCTACCTTCGTGGTTTTACTTGAGTGACG<br>CTGCTCATGCTTGCAACCGCTGGGATGCAGGTGCATGCCTCT |

| | | | |
|---|---|---|---|
| **3360784_C** | TTTGGCCTCCTGAACGTGCGTTGCACTCTTGGGATTTCCTGAGAGTATGTCTGCT<br>TCAGTGCTTAACTTGCCCCAACTTTGCAAGCAGGATGTGTTTCTGCCTTGCGTTC<br>TTATGAGCTATTGCCCTCTGAGCCAATGGCTTGTTAATTGCTTGGTTCTTGCAAA<br>ATGCTTTGCGCGCTGTTATTCAGGTTTCTACCTTCGTGGTTTTACTTGAGTGACG<br>CTGCTCATGCTTGCAACCGCTGGGATGCAGGTGCATGCCTCTA | **C1** | TGGCCTCCTGAACGTGCGTTGCACTCTTGGGATTTCCTGAGAGTATGTCTGCTT<br>CAGTGCTTAACTTGCCCCAACTTTGCAAGCAGGATGTGTTTCTGCCTTGCGTTC<br>TTATGAGCTATTGCCCTCTGAGCCAATGGCTTGTTAATTGCTTGGTTCTTGCAAA<br>ATGCTTTGCGCGCTGTTATTCAGGTTTCTACCTTCGTGGTTTTACTTGAGTGACG<br>CTGCTCATGCTTGCAACCGCTGGGATGCAGGTGCATGCCTCT |
| **702368_C** | TATGGCCTCCTGAACGTGCGTTGCACTCTTGGGATTTCCTGAGAGTATGTCTGCT<br>TCAGTGCTTAACTTGCCCCAACTTTGCAAGCAGGATGTGTTTCTGCCTTGCGTTC<br>TTATGAGCTATTGCCCTCTGAGCCAATGGCTTGTTAATTGCTTCGTTCTTGCAAA<br>ATGCTTTGCGCGCTGTTATTCAGGTTTCTACCTTCGTGGTTTTACTTGAGTGACG<br>CTGCTCATGCTTGCAACCGCTGGGATGCAGGTGCATGCCTCTA | **C1al** | TGGCCTCCTGAACGTGCGTTGCACTCTTGGGATTTCCTGAGAGTATGTCTGCTT<br>CAGTGCTTAACTTGCCCCAACTTTGCAAGCAGGATGTGTTTCTGCCTTGCGTTC<br>TTATGAGCTATTGCCCTCTGAGCCAATGGCTTGTTAATTGCTTCGTTCTTGCAAA<br>ATGCTTTGCGCGCTGTTATTCAGGTTTCTACCTTCGTGGTTTTACTTGAGTGACG<br>CTGCTCATGCTTGCAACCGCTGGGATGCAGGTGCATGCCTCT |
| **C1lq** | AAATGGCCTCCTGAACGTGCGTTGCACTCTTGGGATTTCCTGAGAGTATGTCTGC<br>TTCAGTGCTTAACTTGCCCCAACTTTGCAAGCAGATGTGTTTCTGCCTTGCGTTC<br>TTATGAGCTATTGCCCTCTGAGCCAATGGCTTGTTAATTGCTTGGTTCTTGCAAA<br>ATGCTTTGCGCGCTGTTATTCAGGTTTCTACCTTCGTGGTTTTACTTGAGTGACG<br>CTGCTCATGCTTGCAACCGCTGGGATGCAGGTGCATGCCTCT | **C1b** | TGGCCTCCTGAACGTGCGTTGCACTCTTGGGATTTCCTGAGAGTATGTCTGCTT<br>CAGTGCTTAACTTGCCCCAACTTTGCAAGCAGATGTGTTTCTGCCTTGCGTTCT<br>TATGAGCTATTGCCCTCTGAGCCAATGGCTTGTTAATTGCTTGGTTCTTGCAAAA<br>TGCTTTGCGCGCTGTTATTCAGGTTTCTACCTTCGTGGTTTTACTTGAGTGACGC<br>TGCTCATGCTTGCAACCGCTGGGATGCAGGTGCATGCCTCT |
| **C1ot** | TATGGCCTCCTGAACGTGCGTTGCACTCTTGGGATTTCCTGAGAGTATGTCTGCT<br>TCAGTGCTTAACTTGCCCCAACTTTGCAAGCAGATGTGTTTCTGCCTTGCGTTCT<br>TATGAGCTATTGCCCTCTGAGCCAATGGCTTGTTAATTGCTTGGTTCTTGCAAAAT<br>GCTTTGCGCGCTGTTATTCAGGTTTCTACCTTCGTGGTTTTACTTGAGTGACGCT<br>GCTCATGCTTGCAACCGCTGGGATGCAGGTGCATGCCTCTA | **C1b** | TGGCCTCCTGAACGTGCGTTGCACTCTTGGGATTTCCTGAGAGTATGTCTGCTT<br>CAGTGCTTAACTTGCCCCAACTTTGCAAGCAGATGTGTTTCTGCCTTGCGTTCT<br>TATGAGCTATTGCCCTCTGAGCCAATGGCTTGTTAATTGCTTGGTTCTTGCAAAA<br>TGCTTTGCGCGCTGTTATTCAGGTTTCTACCTTCGTGGTTTTACTTGAGTGACGC<br>TGCTCATGCTTGCAACCGCTGGGATGCAGGTGCATGCCTCT |
| **C1ov** | TATGGCCTCCTGAACGTGCGTTGCACTCTTGGGATTTCCTGAGAGTATGTCTGCT<br>TCAGTGCTTAACTTGCCCCAACTTTGCAAGCAGATGTGTTTCTGCCTTGCGTTCT<br>TATGAGCTATTGCCCTCTGAGCCAATGGCTTGTTAATTGCTTGGTTCTTGCAAAAT<br>GCTTTGCGCGCTGTTATTCAGGTTTCTACCTTCGTGGTTTTACTTGAGTAACGCT<br>GCTCATGCTTGCAACCGCTGGGATGCAGGTGCATGCCTCTA | **C1bh** | TGGCCTCCTGAACGTGCGTTGCACTCTTGGGATTTCCTGAGAGTATGTCTGCTT<br>CAGTGCTTAACTTGCCCCAACTTTGCAAGCAGATGTGTTTCTGCCTTGCGTTCT<br>TATGAGCTATTGCCCTCTGAGCCAATGGCTTGTTAATTGCTTGGTTCTTGCAAAA<br>TGCTTTGCGCGCTGTTATTCAGGTTTCTACCTTCGTGGTTTTACTTGAGTAACGC<br>TGCTCATGCTTGCAACCGCTGGGATGCAGGTGCATGCCTCT |
| **C1qo** | TATGGCCTCCTGAACGTGCGTTGCACTCTTGGGATTTCCTGAGAGTATGTCTGCT<br>TCAGTGCTTAACTTGCCCCAACTTTGCAAGCAGGATGTGTTTCTGCCTTGCGTTC<br>TTATGAACTATTGCCCTCTGAGCCAATGGCTTGTTAATTGCTTGGTTCTTGCAAA<br>ATGCTTTGCGCGCTGTTATTCAGGTTTCTACCTTCGTGGTTTTACTTGAGTGACG<br>CTGCTCATGCTTGCAACCGCTGGGATGCAGGTGCATGCCTCTA | **C1br** | TGGCCTCCTGAACGTGCGTTGCACTCTTGGGATTTCCTGAGAGTATGTCTGCTT<br>CAGTGCTTAACTTGCCCCAACTTTGCAAGCAGGATGTGTTTCTGCCTTGCGTTC<br>TTATGAACTATTGCCCTCTGAGCCAATGGCTTGTTAATTGCTTGGTTCTTGCAAA<br>ATGCTTTGCGCGCTGTTATTCAGGTTTCTACCTTCGTGGTTTTACTTGAGTGACG<br>CTGCTCATGCTTGCAACCGCTGGGATGCAGGTGCATGCCTCT |
| **C1ou** | TATGGCCTCCTGAATGTGCGTTGCACTCTTGGGATTTCCTGAGAGTATGTCTGCT<br>TCAGTGCTTAACTTGCCCCAACTTTGCAAGCAGGATGTGTTTCTGCCTTGCGTTC<br>TTATGAGCTATTGCCCTCTGAGCCAATGGCTTGTTAATTGCTTGGTTCTTGCAAA<br>ATGCTTTGCGCGCTGTTATTCAGGTTTCTACCTTCGTGGTTTTACTTGAGTGACG<br>CTGCTCATGCTTGCAACCGCTGGGATGCAGGTGCATGCCTCTA | **C1c** | TGGCCTCCTGAATGTGCGTTGCACTCTTGGGATTTCCTGAGAGTATGTCTGCTT<br>CAGTGCTTAACTTGCCCCAACTTTGCAAGCAGGATGTGTTTCTGCCTTGCGTTC<br>TTATGAGCTATTGCCCTCTGAGCCAATGGCTTGTTAATTGCTTGGTTCTTGCAAA<br>ATGCTTTGCGCGCTGTTATTCAGGTTTCTACCTTCGTGGTTTTACTTGAGTGACG<br>CTGCTCATGCTTGCAACCGCTGGGATGCAGGTGCATGCCTCT |
| **411637_C** | TATGGCCTCCTGAACGTGCGTTGCACTCTTGGGATTTCCTGAGAGTATGTCTGCT<br>TCAGTGCTTAACTTGCCCCAACTTTGCAAACAGATGTGTTTCTGCCTTGCGTTCT | **C1cb** | TGGCCTCCTGAACGTGCGTTGCACTCTTGGGATTTCCTGAGAGTATGTCTGCTT<br>CAGTGCTTAACTTGCCCCAACTTTGCAAACAGATGTGTTTCTGCCTTGCGTTCT |

| | | | |
|---|---|---|---|
| | TATGAGCTATTGCCCTCTGAGCCAATGGCTTGTTAATTGCTTGGTTCTTGCAAAAT<br>GCTTTGCGCGCTGTTATTCAGGTTTCTACCTTCGTGGTTTTACTTGAGTGACGCT<br>GCTCATGCTTGCAACCGCTGGGATGCAGGTGCATGCCTCTA | | TATGAGCTATTGCCCTCTGAGCCAATGGCTTGTTAATTGCTTGGTTCTTGCAAAA<br>TGCTTTGCGCGCTGTTATTCAGGTTTCTACCTTCGTGGTTTTACTTGAGTGACGC<br>TGCTCATGCTTGCAACCGCTGGGATGCAGGTGCATGCCTCT |
| **914659_C** | TATGGCCTCCTGAACGTGCGTTGCACTCTTGGGATTTCCTGAGAGTATGTCTGCT<br>TCAGTGCTTAACTTGCCCCAACTTTGCAAGCAGGATGTGTTTCTGCCTTGCGTTC<br>TTATGAGCTATTGCCCTCTGAGCCAATGGCTTGTTAATTGCTTGGTTCTTGCAAA<br>ATGCTTTGCGCGCTGTTATTCAGGTTTCTACCTTCGTGGTTTTACTTGAGTAACGC<br>TGCTCATGCTTGCAACCGCTGGGATGCAGGTGCATGCCTCTA | **C1cv** | TGGCCTCCTGAACGTGCGTTGCACTCTTGGGATTTCCTGAGAGTATGTCTGCTT<br>CAGTGCTTAACTTGCCCCAACTTTGCAAGCAGGATGTGTTTCTGCCTTGCGTTC<br>TTATGAGCTATTGCCCTCTGAGCCAATGGCTTGTTAATTGCTTGGTTCTTGCAAA<br>ATGCTTTGCGCGCTGTTATTCAGGTTTCTACCTTCGTGGTTTTACTTGAGTAACG<br>CTGCTCATGCTTGCAACCGCTGGGATGCAGGTGCATGCCTCT |
| **184772_C** | TATGGCCTCCTGAACGTGCGTTGCACTCTTGGGATTTCCTGAGAGTATGTCTGCT<br>TCAGTGCTTAACTTGCCCCAACTTTGCAAGCAGGATGTGTTTCTGCCTTGCGTTC<br>TTATGAGCTATTGCCCTCTGAGCCAATGGCTTGTTAATTGCTTGGTTCTTGCAAA<br>ATGCTTTGCGCGCTGTTATTCAGGTTTCTACCTTTGTGGTTTTACTTGAGTGACGC<br>TGCTCATGCTTGCAACCGCTGGGATGCAGGTGCATGCCTCTA | **C1w** | TGGCCTCCTGAACGTGCGTTGCACTCTTGGGATTTCCTGAGAGTATGTCTGCTT<br>CAGTGCTTAACTTGCCCCAACTTTGCAAGCAGGATGTGTTTCTGCCTTGCGTTC<br>TTATGAGCTATTGCCCTCTGAGCCAATGGCTTGTTAATTGCTTGGTTCTTGCAAA<br>ATGCTTTGCGCGCTGTTATTCAGGTTTCTACCTTTGTGGTTTTACTTGAGTGACG<br>CTGCTCATGCTTGCAACCGCTGGGATGCAGGTGCATGCCTCT |
| **C3yn** | TATGGCCTCCTGAACGTGCGTTGCACTCTTGGGATTTCCTGAGAGTATGTCTGCT<br>TCAGTGCTTAACTTGCCCCAACTTTGCAAGCAGGATGTGTTTCTGCCTTGCGTTC<br>TTATGAGCTATTGCCCTCTGAGCCAATGGCTTGTTAATTGCTTGGTTCTTGCAAA<br>ATGCTTTGCGCGCTGTTATTCAAGTTTCTACCTTCGTGGTTTTACTTGAGTGACG<br>CTGCTCATGCTTGCAACCGCTGGGATGCAGGTGCATGCCTCTA | **C3** | TGGCCTCCTGAACGTGCGTTGCACTCTTGGGATTTCCTGAGAGTATGTCTGCTT<br>CAGTGCTTAACTTGCCCCAACTTTGCAAGCAGGATGTGTTTCTGCCTTGCGTTC<br>TTATGAGCTATTGCCCTCTGAGCCAATGGCTTGTTAATTGCTTGGTTCTTGCAAA<br>ATGCTTTGCGCGCTGTTATTCAAGTTTCTACCTTCGTGGTTTTACTTGAGTGACG<br>CTGCTCATGCTTGCAACCGCTGGGATGCAGGTGCATGCCTCT |
| **2285174_C** | TATGGCCTCCTGAACGTGCGTTGCACTCTTGGGATTTCCTGAGAGTATGTCTGCT<br>TCAGTGCTTAACTTGCCCCAACTTTGCAAGCAGGATGTGTTTCTGCCTTGCGTTC<br>TTATGAGCTATTGCCCTCAGCCAATGGCTTGTTAATTGCTTGGTTCTTGCAAAATG<br>CTTTGCGCGCTGTTATTCAAGTTTCTACCTTCGTGGTTTTACTTGAGTGACGCTG<br>CTCATGCTTGCAACCGCTGGGATGCAGGTGCATGCCTCTA | **C3cy** | TGGCCTCCTGAACGTGCGTTGCACTCTTGGGATTTCCTGAGAGTATGTCTGCTT<br>CAGTGCTTAACTTGCCCCAACTTTGCAAGCAGGATGTGTTTCTGCCTTGCGTTC<br>TTATGAGCTATTGCCCTCAGCCAATGGCTTGTTAATTGCTTGGTTCTTGCAAAAT<br>GCTTTGCGCGCTGTTATTCAAGTTTCTACCTTCGTGGTTTTACTTGAGTGACGCT<br>GCTCATGCTTGCAACCGCTGGGATGCAGGTGCATGCCTCT |
| **405073_C** | TATGGCCTCCTGAACGTGCGTTGCACTCTTGGGATTTCCTGAGAGTATGTCTGCT<br>TCAGTGCTTAACTTGCCCCAACTTTGCAAGCAGGATGTGTTTCTGCCTTGCGTTC<br>TTATGAGCTATTGCCCTCTGAGCCAATGGCTTGTTAATTGCTTGGTTCTTGCAAA<br>ATGCTTTGCGCGCCGTTATTCAAGTTTCTACCTTCGTGGTTTTACTTGAGTGACG<br>CTGCTCATGCTTGCAACCGCTGGGATGCAGGTGCATGCCTCTA | **C42ca** | TGGCCTCCTGAACGTGCGTTGCACTCTTGGGATTTCCTGAGAGTATGTCTGCTT<br>CAGTGCTTAACTTGCCCCAACTTTGCAAGCAGGATGTGTTTCTGCCTTGCGTTC<br>TTATGAGCTATTGCCCTCTGAGCCAATGGCTTGTTAATTGCTTGGTTCTTGCAAA<br>ATGCTTTGCGCGCCGTTATTCAAGTTTCTACCTTCGTGGTTTTACTTGAGTGACG<br>CTGCTCATGCTTGCAACCGCTGGGATGCAGGTGCATGCCTCT |
| **379888_C** | TATGGCCTCCTGAACGTGCGTTGCACTCTTGGGATTTCCTGAGAGTATGTCTGCT<br>TCAGTGCTTAACTTGCCCCAACTTTGCAAGCAGGATGTGTTTCTGCCTTGCGTTC<br>TTATGAGCTATTGCCCTCTGAGCCAATGGCTTGTTAATTGTTTGGTTCTTGCAAA<br>ATGCTTTGCGCGCTGTTATTCAGGTTTCTACCTTCGTGGTTTTACTTGAGTGACG<br>CTGCTCATGCTTGCAACCGCTGGGATGCAGGTGCATGCCTCTA | **C3ju** | TGGCCTCCTGAACGTGCGTTGCACTCTTGGGATTTCCTGAGAGTATGTCTGCTT<br>CAGTGCTTAACTTGCCCCAACTTTGCAAGCAGGATGTGTTTCTGCCTTGCGTTC<br>TTATGAGCTATTGCCCTCTGAGCCAATGGCTTGTTAATTGTTTGGTTCTTGCAAA<br>ATGCTTTGCGCGCTGTTATTCAGGTTTCTACCTTCGTGGTTTTACTTGAGTGACG<br>CTGCTCATGCTTGCAACCGCTGGGATGCAGGTGCATGCCTCT |
| **1025487_C** | TATGGCCTCCTGAACGTGCGTTGCACTCTTGGGATTTCCTGAGAGTATGTCTGCT<br>TCAGTGCTTAACTTGCCCCAACTTTGCAAGCAGGATGTGTTTCTGCCTTGCGTTC<br>TTATGAGCTATTGCCCTCTGAGCCAATGGCTTGTTAATTGCCTGGTTCTTGCAAA | **C3sa** | TGGCCTCCTGAACGTGCGTTGCACTCTTGGGATTTCCTGAGAGTATGTCTGCTT<br>CAGTGCTTAACTTGCCCCAACTTTGCAAGCAGGATGTGTTTCTGCCTTGCGTTC<br>TTATGAGCTATTGCCCTCTGAGCCAATGGCTTGTTAATTGCCTGGTTCTTGCAAA |

| | | | |
|---|---|---|---|
| | ATGCTTTGCGCGCTGTTATTCAGGTTTCTACCTTCGTGGTTTTACTTGAGTGACG<br>CTGCTCATGCTTGCAACCGCTGGGATGCAGGTGCATGCCTCTA | | ATGCTTTGCGCGCTGTTATTCAGGTTTCTACCTTCGTGGTTTTACTTGAGTGACG<br>CTGCTCATGCTTGCAACCGCTGGGATGCAGGTGCATGCCTCT |
| **C42ep** | TATGGCCTCCTGAACGTGCGTTGCACTCTTGGGATTTCCTGAGAGTATGTCTGCT<br>TCAGTGCTTAACTTGCCCCAACTTTGCAAGCAGGATGTGTTTCTGCCTTGCGTTC<br>TTATGAGCTATTGCCCTCTGAGCCAATGGCTTGTGAATTGCTTGGTTCTTGCAAA<br>ATGCTTTGCGCGCTGTTATTCAGGTTTCTACCTTCGTGGTTTTACTTGAGTGACG<br>CTGCTCATGCTTGCAACCGCTGGGATGCAGGTGCATGCCTCTA | **C42.2** | TGGCCTCCTGAACGTGCGTTGCACTCTTGGGATTTCCTGAGAGTATGTCTGCTT<br>CAGTGCTTAACTTGCCCCAACTTTGCAAGCAGGATGTGTTTCTGCCTTGCGTTC<br>TTATGAGCTATTGCCCTCTGAGCCAATGGCTTGTGAATTGCTTGGTTCTTGCAAA<br>ATGCTTTGCGCGCTGTTATTCAGGTTTCTACCTTCGTGGTTTTACTTGAGTGACG<br>CTGCTCATGCTTGCAACCGCTGGGATGCAGGTGCATGCCTCT |
| **985469_C** | TATGGCCTCCTGAACGTGCGTTGCACTCTTGGGATTTCCTGAGAGTATGTCTGCT<br>TCAGTGCTTAACTTGCCCCAACTTTGCAAGCAGATGTGTTTCTGCCTTGCGTTCT<br>TATGAGCTATTGCCCTCTGAGCCAATGGCTTGTGAATTGCTTGGTTCTTGCAAAA<br>TGCTTTGCGCGCTGTTATTCAGGTTTCTACCTTCGTGGTTTTACTTGAGTGACGC<br>TGCTCATGCTTGCAACCGCTGGGATGCAGGTGCATGCCTCTA | **C42ao** | TGGCCTCCTGAACGTGCGTTGCACTCTTGGGATTTCCTGAGAGTATGTCTGCTT<br>CAGTGCTTAACTTGCCCCAACTTTGCAAGCAGATGTGTTTCTGCCTTGCGTTCT<br>TATGAGCTATTGCCCTCTGAGCCAATGGCTTGTGAATTGCTTGGTTCTTGCAAA<br>ATGCTTTGCGCGCTGTTATTCAGGTTTCTACCTTCGTGGTTTTACTTGAGTGACG<br>CTGCTCATGCTTGCAACCGCTGGGATGCAGGTGCATGCCTCT |
| **396862_C** | TATGGCCTCCTGAACGTGCGTTGCACTCTTGGGATTTCCTGAGAGTATGTCTGCT<br>TCAGTGCTTAACTTGCCCCAACTTTGCAAGCAGGATGTGTTTCTGCCTTGCGTTC<br>TTATGAGCTGTTGCCAATGGCTTGTTAATTGCTTGGTTCTTGCAAAATGCTTTGC<br>GCGCTGTTATTCAGGTTTCTACCTTCGTGGTTTTACTTGAGTGACGCTGCTCATG<br>CTTGCAACCGCTGGGATGCAGGTGCATGCCTCTA | **C72k** | TGGCCTCCTGAACGTGCGTTGCACTCTTGGGATTTCCTGAGAGTATGTCTGCTT<br>CAGTGCTTAACTTGCCCCAACTTTGCAAGCAGGATGTGTTTCTGCCTTGCGTTC<br>TTATGAGCTGTTGCCAATGGCTTGTTAATTGCTTGGTTCTTGCAAAATGCTTTGC<br>GCGCTGTTATTCAGGTTTCTACCTTCGTGGTTTTACTTGAGTGACGCTGCTCATG<br>CTTGCAACCGCTGGGATGCAGGTGCATGCCTCT |

**Table S2. Experimental sample sizes.** Number of slides and live recruits produced and studied are provided for each offspring group from crosses amongst the most sensitive (SS), moderately resilient (MM), and most resilient (RR) broodstock colonies, and a bulk cross (Bulk) produced from all broodstock gametes spawned in synchrony. The number of broodstock colonies whose gametes contributed to each of the crosses is also listed.

| **Reef** | **Offspring group** | **Broodstock (*n*)** | **Slides (*n*)** | **Live recruits (*n*)** |
|---|---|---|---|---|
| Davies | SS | 3 | 64 | 203 |
| Davies | MM | 3 | 32 | 136 |
| Davies | RR | 3 | 78 | 371 |
| Davies | Bulk | 9 | 36 | 142 |
| Moore | SS | 3 | 41 | 207 |
| Moore | MM | 4 | 45 | 165 |
| Moore | RR | 3 | 37 | 164 |
| Moore | Bulk | 6 | 44 | 168 |

**Table S3. Primary extraction buffer (PEB) reagents.** The volume and final concentration of each reagent required to generate 75 mL of PEB is listed.

| Stock Solution | Stock to add for 75 mL (~100 samples) | Final concentration |
|---|---|---|
| Milli Q | 43.5 mL | Milli Q |
| 1 M Tris pH 9.0 | 7.5 mL | 100 mM Tris pH 9.0 |
| 500 mM EDTA | 15.0 mL | 100 mM EDTA |
| 5 M NaCl | 1.5 mL | 100 mM NaCl |
| 10% SDS (add last) | 7.5 mL | 1 % SDS |

**Table S4. DNA extraction reagents in addition to those required for the primary extraction buffer**. The volume of each reagent required to extract ~ 100 samples is listed.

| Stock Solution | Stock required for ~100 samples |
|---|---|
| Lysozyme (10mg/mL) | 507 µL |
| Proteinase K (20mg/mL) | 2.0 mL |
| *5 M Potassium acetate (KoAC) | 20.65 mL |
| *100% Isopropanol | 71.25 mL |
| *70% Ethanol (EtOH) | 22.5 mL |

* *These volumes will alter with the number of samples being processed – the values allow for the maximum number of samples that can be extracted with the indicated volume (75 mL) of extraction buffer and a small "pipetting error' safety net.*

** *Adjust the reagent volumes as appropriate for the number of samples to be processed. If tissue is small, for example single coral recruits, consider using half the volume of primary extraction buffer and reagents per sample tube.*

## REFERENCES


1. IPCC, "*Climate Change 2022: Impacts, Adaptation, and Vulnerability. Contribution of Working Group II to the Sixth Assessment Report of the Intergovernmental Panel on Climate Change*" (Cambridge Univ. Press, 2022).

2. C. M. Sgro, A. J. Lowe, A. A. Hoffmann, Building evolutionary resilience for conserving biodiversity under climate change. *Evol. Appl.* **4**, 326–337 (2011).

3. A. D. Barnosky, N. Matzke, S. Tomiya, G. O. Wogan, B. Swartz, T. B. Quental, C. Marshall, J. L. McGuire, E. L. Lindsey, K. C. Maguire, Has the Earth's sixth mass extinction already arrived? *Nature* **471**, 51–57 (2011).

4. J. M. De Vos, L. N. Joppa, J. L. Gittleman, P. R. Stephens, S. L. Pimm, Estimating the normal background rate of species extinction. *Conserv. Biol.* **29**, 452–462 (2015).

5. R. Warren, J. VanDerWal, J. Price, J. A. Welbergen, I. Atkinson, J. Ramirez-Villegas, T. J. Osborn, A. Jarvis, L. P. Shoo, S. E. Williams, Quantifying the benefit of early climate change mitigation in avoiding biodiversity loss. *Nat. Clim. Change* **3**, 678–682 (2013).

6. A. R. Weeks, D. Heinze, L. Perrin, J. Stoklosa, A. A. Hoffmann, A. van Rooyen, T. Kelly, I. Mansergh, Genetic rescue increases fitness and aids rapid recovery of an endangered marsupial population. *Nat. Commun.* **8**, 1071 (2017).

7. J. A. Hamilton, J. M. Miller, Adaptive introgression as a resource for management and genetic conservation in a changing climate. *Conserv. Biol.* **30**, 33–41 (2016).

8. A. Pavlova, S. Petrovic, K. A. Harrisson, K. Cartwright, E. Dobson, L. L. Hurley, M. Lane, M. J. Magrath, K. A. Miller, B. Quin, Benefits of genetic rescue of a critically endangered subspecies from another subspecies outweigh risks: Results of captive breeding trials. *Biol. Conserv.* **284**, 110203 (2023).

9. S. M. Carlson, C. J. Cunningham, P. A. Westley, Evolutionary rescue in a changing world. *Trends Ecol. Evol.* **29**, 521–530 (2014).

10. T. A. Jones, T. A. Monaco, A role for assisted evolution in designing native plant materials for domesticated landscapes. *Front. Ecol. Environ.* **7**, 541–547 (2009).

11. M. J. H. van Oppen, R. D. Gates, L. L. Blackall, N. Cantin, L. J. Chakravarti, W. Y. Chan, C. Cormick, A. Crean, K. Damjanovic, H. Epstein, P. L. Harrison, T. A. Jones, M. Miller, R. J. Pears, L. M. Peplow, D. A. Raftos, B. Schaffelke, K. Stewart, G. Torda, D. Wachenfeld, A. R. Weeks, H. M. Putnam, Shifting paradigms in restoration of the world's coral reefs. *Glob. Chang. Biol.* **23**, 3437–3448 (2017).

12. National Academies of Sciences Engineering and Medicine, *A Research Review of Interventions to Increase the Persistence and Resilience of Coral Reefs* (National Academies Press, 2019).

13. M. J. H. van Oppen, J. K. Oliver, H. M. Putnam, R. D. Gates, Building coral reef resilience through assisted evolution. *Proc. Natl. Acad. Sci. U.S.A.* **112**, 2307–2313 (2015).

14. D. Souter, S. Planes, J. Wicquart, M. Logan, D. Obura, F. Staub, "Status of coral reefs of the world: 2020" (2021).

15. O. Hoegh-Guldberg, Climate change, coral bleaching and the future of the world's coral reefs. *Mar. Freshw. Res.* **50**, 839–866 (1999).

16. Y. M. Bozec, K. Hock, R. A. Mason, M. E. Baird, C. Castro-Sanguino, S. A. Condie, M. Puotinen, A. Thompson, P. J. Mumby, Cumulative impacts across Australia's Great Barrier Reef: A mechanistic evaluation. *Ecological monographs* **92**, e01494 (2022).

17. M. W. Cheung, K. Hock, W. Skirving, P. J. Mumby, Cumulative bleaching undermines systemic resilience of the Great Barrier Reef. *Curr. Biol.* **31**, 5385–5392.e4 (2021).

18. Australian Institute of Marine Science Long-Term Monitoring Program, "Great Barrier Reef Annual Summary Report Coral Reef Condition 2024/2025" (2025).

19. A. M. Dixon, P. M. Forster, S. F. Heron, A. M. Stoner, M. Beger, Future loss of local-scale thermal refugia in coral reef ecosystems. *PLOS Climate* **1**, e0000004 (2022).

20. C. J. Randall, A. P. Negri, K. M. Quigley, T. Foster, G. F. Ricardo, N. S. Webster, L. K. Bay, P. L. Harrison, R. C. Babcock, A. J. Heyward, Sexual production of corals for reef restoration in the Anthropocene. *Mar. Ecol. Prog. Ser.* **635**, 203–232 (2020).

21. L. K. Bay, J. Gilmour, B. Muir, P. E. Hardisty, Management approaches to conserve Australia's marine ecosystem under climate change. *Science* **381**, 631–636 (2023).

22. C. Caruso, K. Hughes, C. Drury, Selecting heat-tolerant corals for proactive reef restoration. *Front. Mar. Sci.* **8**, 584 (2021).

23. A. Humanes, L. Lachs, E. Beauchamp, L. Bukurou, D. Buzzoni, J. Bythell, J. R. Craggs, R. de la Torre Cerro, A. J. Edwards, Y. Golbuu, Selective breeding enhances coral heat tolerance to marine heatwaves. *Nat. Commun.* **15**, 8703 (2024).

24. D. N. Duvick, The contribution of breeding to yield advances in maize (*Zea mays L.*). *Adv. Agron.* **86**, 83–145 (2005).

25. A. E. Hall, *Breeding for Heat Tolerance. Plant Breeding Reviews* (John Wiley & Sons, 1992), vol. 10, pp. 129–168.

26. C. Lind, R. Ponzoni, N. H. Nguyen, H. Khaw, Selective breeding in fish and conservation of genetic resources for aquaculture. *Reprod. Domest. Anim.* **47**, 255–263 (2012).

27. C. Layton, C. R. Johnson, Assessing the feasibility of restoring giant kelp forests in Tasmania (National Environmental Science Program, Marine Biodiversity Hub, 2021).

28. M. E. Jones, P. J. Jarman, C. M. Lees, H. Hesterman, R. K. Hamede, N. J. Mooney, D. Mann, C. E. Pukk, J. Bergfeld, H. McCallum, Conservation management of Tasmanian devils in the context of an emerging, extinction-threatening disease: Devil facial tumor disease. *Ecohealth* **4**, 326–337 (2007).

29. R. A. Bay, S. R. Palumbi, Multilocus adaptation associated with heat resistance in reef-building corals. *Curr. Biol.* **24**, 2952–2956 (2014).

30. Z. L. Fuller, V. J. L. Mocellin, L. A. Morris, N. Cantin, J. Shepherd, L. Sarre, J. Peng, Y. Liao, J. Pickrell, P. Andolfatto, M. Matz, L. K. Bay, M. Przeworski, Population genetics of the coral *Acropora millepora*: Toward genomic prediction of bleaching. *Science* **369**, eaba4674 (2020).

31. C. Drury, N. K. Bean, C. I. Harris, J. R. Hancock, J. Huckeba, C. Martin H, T. N. F. Roach, R. A. Quinn, R. D. Gates, Intrapopulation adaptive variance supports thermal tolerance in a reef-building coral. *Commun. Biol.* **5**, 486 (2022).

32. C. R. Voolstra, C. Buitrago-Lopez, G. Perna, A. Cardenas, B. C. C. Hume, N. Radecker, D. J. Barshis, Standardized short-term acute heat stress assays resolve historical differences in coral thermotolerance across microhabitat reef sites. *Glob. Chang. Biol.* **26**, 4328–4343 (2020).

33. M. R. Marzonie, L. K. Bay, D. G. Bourne, A. S. Hoey, S. Matthews, J. J. Nielsen, H. B. Harrison, The effects of marine heatwaves on acute heat tolerance in corals. *Glob. Chang. Biol.* **29**, 404–416 (2023).

34. M. S. Naugle, H. Denis, V. J. Mocellin, P. W. Laffy, I. Popovic, L. K. Bay, E. J. Howells, Heat tolerance varies considerably within a reef-building coral species on the Great Barrier Reef. *Commun. Earth Environ.* **5**, 525 (2024).

35. N. R. Evensen, C. R. Voolstra, M. Fine, G. Perna, C. Buitrago-López, A. Cárdenas, G. Banc-Prandi, K. Rowe, D. J. Barshis, Empirically derived thermal thresholds of four coral species along the Red Sea using a portable and standardized experimental approach. *Coral Reefs* **41**, 239–252 (2022).

36. L. J. Chakravarti, V. H. Beltran, M. J. H. van Oppen, Rapid thermal adaptation in photosymbionts of reef-building corals. *Glob. Chang. Biol.* **23**, 4675–4688 (2017).

37. H. J. Scharfenstein, C. Alvarez-Roa, L. M. Peplow, P. Buerger, W. Y. Chan, M. J. van Oppen, Chemical mutagenesis and thermal selection of coral photosymbionts induce adaptation to heat stress with trait trade-offs. *Evol. Appl.* **16**, 1549–1567 (2023).

38. L. J. Chakravarti, M. J. H. van Oppen, Experimental evolution in coral photosymbionts as a tool to increase thermal tolerance. *Front. Mar. Sci.* **5**, s (2018).

39. H. J. Scharfenstein, L. M. Peplow, C. Alvarez-Roa, M. R. Nitschke, W. Y. Chan, P. Buerger, M. J. H. van Oppen, Pushing the limits: Expanding the temperature tolerance of a coral photosymbiont through differing selection regimes. *New Phytol.* **243**, 2130–2145 (2024).

40. I. E. Huertas, M. Rouco, V. Lopez-Rodas, E. Costas, Warming will affect phytoplankton differently: Evidence through a mechanistic approach. *Proc. R. Soc. B Biol. Sci.* **278**, 3534–3543 (2011).

41. D. Abrego, K. E. Ulstrup, B. L. Willis, M. J. van Oppen, Species–specific interactions between algal endosymbionts and coral hosts define their bleaching response to heat and light stress. *Proc. R. Soc. B Biol. Sci.* **275**, 2273–2282 (2008).

42. P. Buerger, C. Alvarez-Roa, C. W. Coppin, S. L. Pearce, L. J. Chakravarti, J. G. Oakeshott, O. R. Edwards, M. J. H. van Oppen, Heat-evolved microalgal symbionts increase coral bleaching tolerance. *Sci. Adv.* **6**, eaba2498 (2020).

43. W. Y. Chan, L. Meyers, D. Rudd, S. H. Topa, M. J. van Oppen, Heat-evolved algal symbionts enhance bleaching tolerance of adult corals without trade-off against growth. *Glob. Chang. Biol.* **29**, 6945–6968 (2023).

44. K. M. Quigley, C. Alvarez-Roa, J.-B. Raina, M. Pernice, M. J. van Oppen, Heat-evolved microalgal symbionts increase thermal bleaching tolerance of coral juveniles without a trade-off against growth. *Coral Reefs* **42**, 1227–1232 (2023).

45. M. R. Nitschke, D. Abrego, C. E. Allen, C. Alvarez-Roa, N. M. Boulotte, P. Buerger, W. Y. Chan, W. A. F. Neto, E. Ivory, B. Johnston, The use of experimentally evolved coral photosymbionts for reef restoration. *Trends Microbiol.* **32**, 1241–1252 (2024).

46. H. Denis, L. K. Bay, V. J. L. Mocellin, M. S. Naugle, G. Lecellier, S. W. Purcell, V. Berteaux-Lecellier, E. J. Howells, Thermal tolerance traits of individual corals are widely distributed across the Great Barrier Reef. *Proc. R. Soc. B* **291**, 20240587 (2024).

47. National Oceanic and Atmospheric Administration Coral Reef Watch. (2021), vol. 2021.

48. R. V. Lenth. (2022).

49. J. K. McWhorter, P. R. Halloran, G. Roff, W. J. Skirving, C. T. Perry, P. J. Mumby, The importance of 1.5° C warming for the Great Barrier Reef. *Glob. Chang. Biol.* **28**, 1332–1341 (2022).

50. A. M. Lamb, L. M. Peplow, P. L. Harrison, C. A. Humphrey, L. Latini, G. A. McCutchan, M. J. van Oppen, Coral recruits demonstrate thermal resilience. *PeerJ* **12**, e18273 (2024).

51. K. L. Tietjen, N. F. Perks, N. C. O'Brien, J. K. Baum, Impacts of a prolonged marine heatwave and chronic local human disturbance on juvenile coral assemblages. *PLOS ONE* **20**, e0300084 (2025).

52. National Oceanic and Atmospheric Administration Coral Reef Watch. (2025), vol. 2025.

53. A. C. Baker, P. W. Glynn, B. Riegl, Climate change and coral reef bleaching: An ecological assessment of long-term impacts, recovery trends and future outlook. *Estuar. Coast. Shelf Sci.* **80**, 435–471 (2008).

54. G. F. Ricardo, C. E. Harper, A. P. Negri, H. M. Luter, M. A. A. Wahab, R. J. Jones, Impacts of water quality on Acropora coral settlement: The relative importance of substrate quality and light. *Sci. Total Environ.* **777**, 146079 (2021).

55. C. Doropoulos, G. Roff, Y. M. Bozec, M. Zupan, J. Werminghausen, P. J. Mumby, Characterizing the ecological trade-offs throughout the early ontogeny of coral recruitment. *Ecol. Monogr.* **86**, 20–44 (2016).

56. S. Hazraty-Kari, P. Tavakoli-Kolour, S. Kitanobo, T. Nakamura, M. Morita, Adaptations by the coral Acropora tenuis confer resilience to future thermal stress. *Commun. Biol.* **5**, 1371 (2022).

57. M. Yorifuji, S. Harii, R. Nakamura, M. Fudo, Shift of symbiont communities in Acropora tenuis juveniles under heat stress. *PeerJ* **5**, e4055 (2017).

58. K. M. Quigley, C. J. Randall, M. J. H. van Oppen, L. K. Bay, Assessing the role of historical temperature regime and algal symbionts on the heat tolerance of coral juveniles. *Biol. Open* **9**, bio047316 (2020).

59. N. R. Evensen, M. Fine, G. Perna, C. R. Voolstra, D. J. Barshis, Remarkably high and consistent tolerance of a Red Sea coral to acute and chronic thermal stress exposures. *Limnol. Oceanogr.* **66**, 1718–1729 (2021).

60. S. Haider, J. Iqbal, S. Naseer, T. Yaseen, M. Shaukat, H. Bibi, Y. Ahmad, H. Daud, N. L. Abbasi, T. Mahmood, Molecular mechanisms of plant tolerance to heat stress: current landscape and future perspectives. *Plant Cell Rep.* **40**, 2247–2271 (2021).

61. O. S. McCarthy, M. Winston Pomeroy, J. E. Smith, Corals that survive repeated thermal stress show signs of selection and acclimatization. *PLOS ONE* **19**, e0303779 (2024).

62. M. Ruggeri, Y. Zhang, G. V. Aglyamova, C. D. Kenkel, Divergent transcriptional response to thermal stress among life stages could constrain coral adaptation to climate change. *Front. Mar. Sci.* **10**, 1163552 (2023).

63. M. A. Albecker, L. G. Wilkins, S. A. Krueger-Hadfield, S. M. Bashevkin, M. W. Hahn, M. P. Hare, H. K. Kindsvater, M. A. Sewell, K. E. Lotterhos, A. M. Reitzel, Does a complex life cycle affect adaptation to environmental change? Genome-informed insights for characterizing selection across complex life cycle. *Proc. R. Soc. B* **288**, 20212122 (2021).

64. A. Macadam, C. Morgans, J. Cheok, K. Damjanovic, M. Ciampaglia, M. Toor, P. Laffy, I. R. Cooke, J. M. Strugnell, K. M. Quigley, Assessing the potential for "assisted gene flow" to enhance heat tolerance of multiple coral genera over three key phenotypic traits. *Biol. Conserv.* **306**, 111155 (2025).

65. T. C. L. Bridge, P. F. Cowman, A. M. Quattrini, V. E. Bonito, F. Sinniger, S. Harii, C. E. I. Head, J. Y. Hung, T. Halafihi, T. Rongo, A. H. Baird, A tenuis relationship: Traditional taxonomy obscures systematics and biogeography of the '*Acropora tenuis*' (Scleractinia: Acroporidae) species complex. *Zool. J. Linn. Soc.* **202**, 1–24 (2023).

66. W. Y. Chan, R. Sakamoto, T. Doering, V. K. Narayana, D. P. De Souza, M. J. McConville, M. J. van Oppen, Heat-evolved microalgae (Symbiodiniaceae) are stable symbionts and influence thermal tolerance of the sea anemone exaiptasia diaphana. *Environ. Microbiol.* **27**, e70011 (2025).

67. C. C. Butler, K. E. Turnham, A. M. Lewis, M. R. Nitschke, M. E. Warner, D. W. Kemp, O. Hoegh-Guldberg, W. K. Fitt, M. J. van Oppen, T. C. LaJeunesse, Formal recognition of host-generalist species of dinoflagellate (Cladocopium, Symbiodiniaceae) mutualistic with Indo-Pacific reef corals. *J. Phycol.* **59**, 698–711 (2023).

68. B. D. Ramsby, R. Forster, S. N. Ferguson, P. Haikola, C. J. Randall, M. A. Abdul Wahab, D. J. Mead, A. Severati, Developing coral seeding devices and rapid deployment methods to scale up reef restoration. *Restor. Ecol.* **34**, e70206 (2026).

69. A. Severati, F. M. Nordborg, A. Heyward, M. A. Abdul Wahab, C. A. Brunner, J. Montalvo-Proano, A. P. Negri, The AutoSpawner system–sAutomated ex situ spawning and fertilisation of corals for reef restoration. *J. Environ. Manage.* **366**, 121886 (2024).

70. I. B. Baums, A. C. Baker, S. W. Davies, A. G. Grottoli, C. D. Kenkel, S. A. Kitchen, I. B. Kuffner, T. C. LaJeunesse, M. V. Matz, M. W. Miller, Considerations for maximizing the adaptive potential of restored coral populations in the western Atlantic. *Ecol. Appl.* **29**, e01978 (2019).

71. I. B. Baums, V. F. Chamberland, N. S. Locatelli, T. Conn, in *Coral Reef Conservation and Restoration in the Omics Age* (Springer, 2022), pp. 35–53.

72. C. Young, S. Schopmeyer, D. Lirman, A review of reef restoration and coral propagation using the threatened genus Acropora in the Caribbean and Western Atlantic. *Bull. Mar. Sci.* **88**, 1075–1098 (2012).

73. J. S. Madin, K. D. Anderson, M. H. Andreasen, T. C. Bridge, S. D. Cairns, S. R. Connolly, E. S. Darling, M. Diaz, D. S. Falster, E. C. Franklin, The Coral Trait Database, a curated database of trait information for coral species from the global oceans. *Sci. Data* **3**, 1–22 (2016).

74. J. Fox, S. Weisberg, *An R Companion to Applied Regression* (Sage, Thousand Oaks CA, 3rd Ed., 2019).

75. T. N. Whitman, A. P. Negri, D. G. Bourne, C. J. Randall, Settlement of larvae from four families of corals in response to a crustose coralline alga and its biochemical morphogens. *Sci. Rep.* **10**, 1–10 (2020).

76. P. M. Erwin, A. Szmant, Settlement induction of Acropora palmata planulae by a GLW-amide neuropeptide. *Coral Reefs* **29**, 929–939 (2010).

77. J. R. Hancock, A. R. Barrows, T. C. Roome, A. S. Huffmyer, S. B. Matsuda, N. J. Munk, S. A. Rahnke, C. Drury, Coral husbandry for ocean futures: Leveraging abiotic factors to increase survivorship, growth, and resilience in juvenile *Montipora capitata*. *Mar. Ecol. Prog. Ser.* **657**, 123–133 (2021).

78. S. A. Rahnke, J. R. Hancock, N. J. Munk, C. Caruso, C. Drury, Optimizing sexual reproduction of *Montipora capitata* for restoration: Effects of abiotic conditions and light acclimation on juvenile survival and growth. *Mar. Ecol. Prog. Ser.* **691**, 41–54 (2022).

79. W. Cornwall, Florida coral restoration in hot water. *Science* **383**, 576–577 (2024).

80. K. Wilson, Y. Li, V. Whan, S. Lehnert, K. Byrne, S. Moore, S. Pongsomboon, A. Tassanakajon, G. Rosenberg, E. Ballment, Genetic mapping of the black tiger shrimp Penaeus monodon with amplified fragment length polymorphism. *Aquaculture* **204**, 297–309 (2002).

81. B. C. C. Hume, M. Ziegler, J. Poulain, X. Pochon, S. Romac, E. Boissin, C. de Vargas, S. Planes, P. Wincker, C. R. Voolstra, An improved primer set and amplification protocol with increased specificity and sensitivity targeting the Symbiodinium ITS2 region. *PeerJ* **6**, e4816 (2018).

82. A. M. Dungan, M. J. Van Oppen, L. L. Blackall, Short-term exposure to sterile seawater reduces bacterial community diversity in the sea anemone, *Exaiptasia diaphana*. *Front. Mar. Sci.* **7**, 599314 (2021).

83. B. C. C. Hume, E. G. Smith, M. Ziegler, H. J. M. Warrington, J. A. Burt, T. C. LaJeunesse, J. Wiedenmann, C. R. Voolstra, SymPortal: A novel analytical framework and platform for coral algal symbiont next-generation sequencing ITS2 profiling. *Mol. Ecol. Resour.* **19**, 1063–1080 (2019).

84. H. J. Scharfenstein, W. Y. Chan, P. Buerger, C. Humphrey, M. J. van Oppen, Evidence for de novo acquisition of microalgal symbionts by bleached adult corals. *ISME J.* **16**, 1676–1679 (2022).

85. J. Wiedenmann, C. D'Angelo, M. L. Mardones, S. Moore, C. E. Benkwitt, N. A. Graham, B. Hambach, P. A. Wilson, J. Vanstone, G. Eyal, Reef-building corals farm and feed on their photosynthetic symbionts. *Nature* **620**, 1018–1024 (2023).

86. S. Berg, D. Kutra, T. Kroeger, C. N. Straehle, B. X. Kausler, C. Haubold, M. Schiegg, J. Ales, T. Beier, M. Rudy, Ilastik: Interactive machine learning for (bio) image analysis. *Nat. Methods* **16**, 1226–1232 (2020).

87. A. Macadam, C. J. Nowell, K. Quigley, Machine learning for the fast and accurate assessment of fitness in coral early life history. *Remote Sens.* **13**, 3173 (2021).

88. J. Schindelin, I. Arganda-Carreras, E. Frise, V. Kaynig, M. Longair, T. Pietzsch, S. Preibisch, C. Rueden, S. Saalfeld, B. Schmid, Fiji: An open-source platform for biological-image analysis. *Nat. Methods* **9**, 676–682 (2012).

89. R Core Team, *R Foundation for Statistical Computing* (2021).

90. H. Wickham (Springer-Verlag, 2016).

91. P.-C. Bürkner, brms: An R package for Bayesian multilevel models using Stan. *J. Stat. Softw.* **80**, 1–28 (2017).

92. E. S. Wright, Using DECIPHER v2. 0 to analyze big biological sequence data in R. *The R Journal* **8**, 352–359 (2016).

93. U. Siebeck, D. Logan, N. Marshall. CoralWatch: A flexible coral bleaching monitoring tool for you and your group, in *11th International Coral Reef Symposium* (2008).

94. A. Kassambara, rstatix: Pipe-friendly framework for basic statistical tests. CRAN: Contributed packages (2019); https://cran.r-project.org/web/packages/rstatix/index.html.
